# Chemoselective Metabolomics via a Modular Reactivity‐Encoding Platform

**DOI:** 10.1002/advs.76599

**Published:** 2026-07-17

**Authors:** Xin Tao, Cang‐Man Zhang, Ru‐Jie Yang, Min Ren, Zhuo‐Ling Zhong, Kang‐Hui Wang, Rui‐Xue Hu, Jia‐Yue Liu, Jun‐Yi Zhou, Hou‐Kai Li, Jian‐Bo Wan

**Affiliations:** ^1^ State Key Laboratory of Mechanism and Quality of Chinese Medicine Institute of Chinese Medical Sciences University of Macau Taipa Macao SAR China; ^2^ School of Pharmacy Shanghai University of Traditional Chinese Medicine Shanghai China

**Keywords:** alkyne‐tagged probe, chemoselective metabolomics, click chemistry, reactivity encoding, submetabolome

## Abstract

Understanding disease‐associated metabolic reprogramming requires comprehensive interrogation of the chemically diverse metabolome. However, conventional liquid chromatography‐mass spectrometry (LC‐MS) workflows analyze metabolites in a largely non‐discriminatory manner, resulting in systematic underrepresentation of specific functional and reactivity classes due to heterogeneous ionization efficiencies and matrix interference. Here, we report a chemoselective metabolomics strategy based on a modular reactivity‐encoding platform (MREP) that enables functional group‐resolved stratification of complex metabolomes. Four orthogonally designed alkyne‐tagged probes selectively derivatize carboxyl, carbonyl, amine, and thiol functionalities under compatible conditions. The encoded metabolites are subsequently immobilized via azide‐alkyne cycloaddition onto a unified solid‐phase capture resin, which simultaneously removes matrix components and installs a diagnostic reporter module. This integrated encoding‐capture architecture achieves high reaction orthogonality, near‐quantitative conversion, and robust quantitative performance across structurally diverse metabolites. The resulting triazole derivatives exhibit markedly enhanced ionization efficiencies and generate a universal reporter‐ion, enabling confident submetabolome classification and reconstruction. Application to serum and liver tissues from mice substantially expands the detectable chemical space, yielding 7 208 features and 1 573 annotated metabolites across four functional group‐defined layers. Collectively, this work establishes the MREP framework as a versatile platform for reactivity‐resolved interrogation of complex small‐molecule systems.

## Graphical abstract

Conventional LC–MS underrepresents numerous metabolites due to heterogeneous ionization efficiencies and matrix interference. Here, we introduce a modular reactivity‐encoding platform (MREP) comprising four alkyne‐tagged probes that selectively derivatize key functional groups, followed by unified click‐chemistry capture to eliminate matrix components and install a universal diagnostic reporter‐ion. This strategy enhances signal intensity and enables function‐resolved metabolomic profiling, resulting in the detection of 7 208 features and the annotation of 1 573 metabolites in mouse liver.

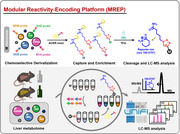

## 1 Introduction

Comprehensive metabolomic profiling is fundamental to understanding biochemical regulation in health and disease. Metabolites function as signaling mediators, enzymatic cofactors, and metabolic intermediates, and their dynamic fluctuations provide direct molecular readouts of cellular states. However, the metabolome constitutes an intrinsically heterogeneous chemical ensemble, encompassing wide variations in polarity, charge state, stability, reactivity, and abundance [1, 2, 3]. Conventional LC‐MS workflows interrogate this diversity in a largely undifferentiated manner, resulting in systematic detection bias toward readily ionizable species while leaving substantial regions of chemical space underexplored [3]. Consequently, current metabolomic analysis is limited not only by sensitivity but also by insufficient chemical resolution.

Chemical derivatization offers a conceptual strategy to impose structural order on this complexity. By selectively transforming defined functional groups, derivatization partitions the metabolome into chemically coherent subsets, often termed submetabolomes [4, 5, 6]. Targeting functional motifs such as carboxyl, carbonyl, amine, and thiol groups enhances ionization efficiency, modulates chromatographic behavior, and introduces characteristic fragmentation signatures. This functional group‐directed “divide‐and‐conquer” strategy has proven effective for improving detection sensitivity and selectivity. Nevertheless, most implementations are limited to single‐class labeling, and the absence of unified capture architectures constrains scalability and multiplexed interrogation across chemically diverse metabolite subsets. Solid‐phase enrichment strategies further enhance selectivity by physically separating derivatized metabolites from complex biological matrices. Reactive functionalities immobilized on resins [6, 7], magnetic beads [8, 9], SiO_2_ nanoprobes [10], and monolithic substrates [11] have enabled chemoselective capture of defined metabolite classes. Despite these advances, existing platforms typically rely on bespoke functionalized supports tailored to individual reactivity classes, requiring multistep synthetic modification and exhibiting limited cross‐compatibility [6, 8, 11]. Such designs restrict versatility and impede parallel stratification of multiple functional groups within a unified framework. A broadly applicable platform that integrates orthogonal chemical encoding with a single, modular capture architecture remains underdeveloped.

Here, we introduce chemoselective metabolomics via a modular reactivity‐encoding platform (MREP) that enables programmable stratification of complex metabolomes based on intrinsic functional group reactivity. Four alkyne‐tagged probes were rationally designed to selectively derivatize carboxyl, carbonyl, amine, and thiol functionalities under compatible conditions. The encoded metabolites are subsequently immobilized through azide‐alkyne cycloaddition onto a unified solid‐phase capture resin engineered for simultaneous enrichment and reporter‐ion installation. Following selective release, the resulting derivatives generate a characteristic diagnostic fragment that enables confident functional‐group assignment and systematic submetabolome reconstruction. This integrated encoding‐capture architecture expands detectable chemical space, minimizes matrix interference, and achieves high reaction efficiency and quantitative robustness across diverse biological matrices. Application to serum and liver tissues from mice demonstrates comprehensive profiling across four functional group‐defined layers, revealing previously obscured metabolic features. Collectively, this work establishes a generalizable chemical platform for structurally resolved metabolomic interrogation.

## 2 Results

### 2.1 Modular Reactivity‐Encoding Platform

**FIGURE 1:**
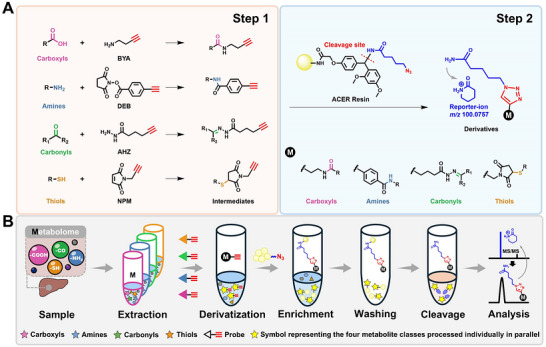
Modular reactivity‐encoding platform for chemoselective metabolomics. (A) Two‐step reactivity‐encoding strategy. In Step 1, alkyne‐tagged probes selectively derivatize metabolites bearing carboxyl, amine, carbonyl, or thiol functional groups to generate encoded intermediates. In Step 2, the intermediates undergo Cu(I) catalyzed azide‐alkyne cycloaddition with ACER resin for covalent capture, followed by acid‐mediated cleavage prior to LC‐MS analysis. (B) Overall analytical workflow enabling functional group‐resolved stratification of complex metabolomes. For clarity, a simplified schematic is presented. Each metabolite class is derivatized and processed individually in parallel workflows, although illustrated here in a unified format.

To enable functional group‐resolved interrogation of complex metabolomes, we established an MREP that integrates orthogonal chemical transformation with unified solid‐phase capture (Figure 1). Rather than directly analyzing heterogeneous metabolite mixtures, this platform imposes reactivity‐based stratification through sequential encoding and enrichment. The MREP architecture comprises two chemically integrated steps. In the encoding step, four alkyne‐tagged probes, but‐3‐ynylamine (BYA), 2,5‐dioxopyrrolidin‐1‐yl 4‐ethynylbenzoate (DEB), alkyne hydrazide (AHZ), and N‐propargylmaleimide (NPM), were rationally designed to selectively derivatize metabolites bearing carboxyl, amine, carbonyl, and thiol functionalities, respectively. Carboxyl and amine metabolites were modified via acylation reaction with BYA and DEB, respectively. Carbonyl metabolites underwent nucleophilic addition with AHZ, whereas thiol metabolites were conjugated through Michael addition with NPM. Each probe incorporates a terminal alkyne, thereby installing a bioorthogonal handle while preserving the native metabolite scaffold. In the capture step, the encoded metabolites are immobilized via Cu(I)‐catalyzed azide‐alkyne cycloaddition onto azide‐functionalized resins, termed alkyne capture for enrichment and reporter‐ion installation (ACER) resins. This click reaction proceeds under mild conditions with high chemoselectivity, generating stable triazole linkages while enabling physical separation from unreacted matrix components. Following extensive washing, the immobilized derivatives are cleaved under acidic conditions and subjected to LC‐MS analysis. Importantly, the resulting triazole‐containing derivatives exhibit substantially enhanced ionization efficiencies relative to their native counterparts. In addition, the embedded valeric amide module produces a diagnostic reporter‐ion at *m/z *100.0757 in MS/MS spectra, providing a universal fragmentation signature across all four functional group‐defined layers. Together, these features transform complex metabolite mixtures into structurally encoded, functionally stratified subsets suitable for confident classification and quantitative analysis.

### 2.2 Optimization of MREP

**FIGURE 2:**
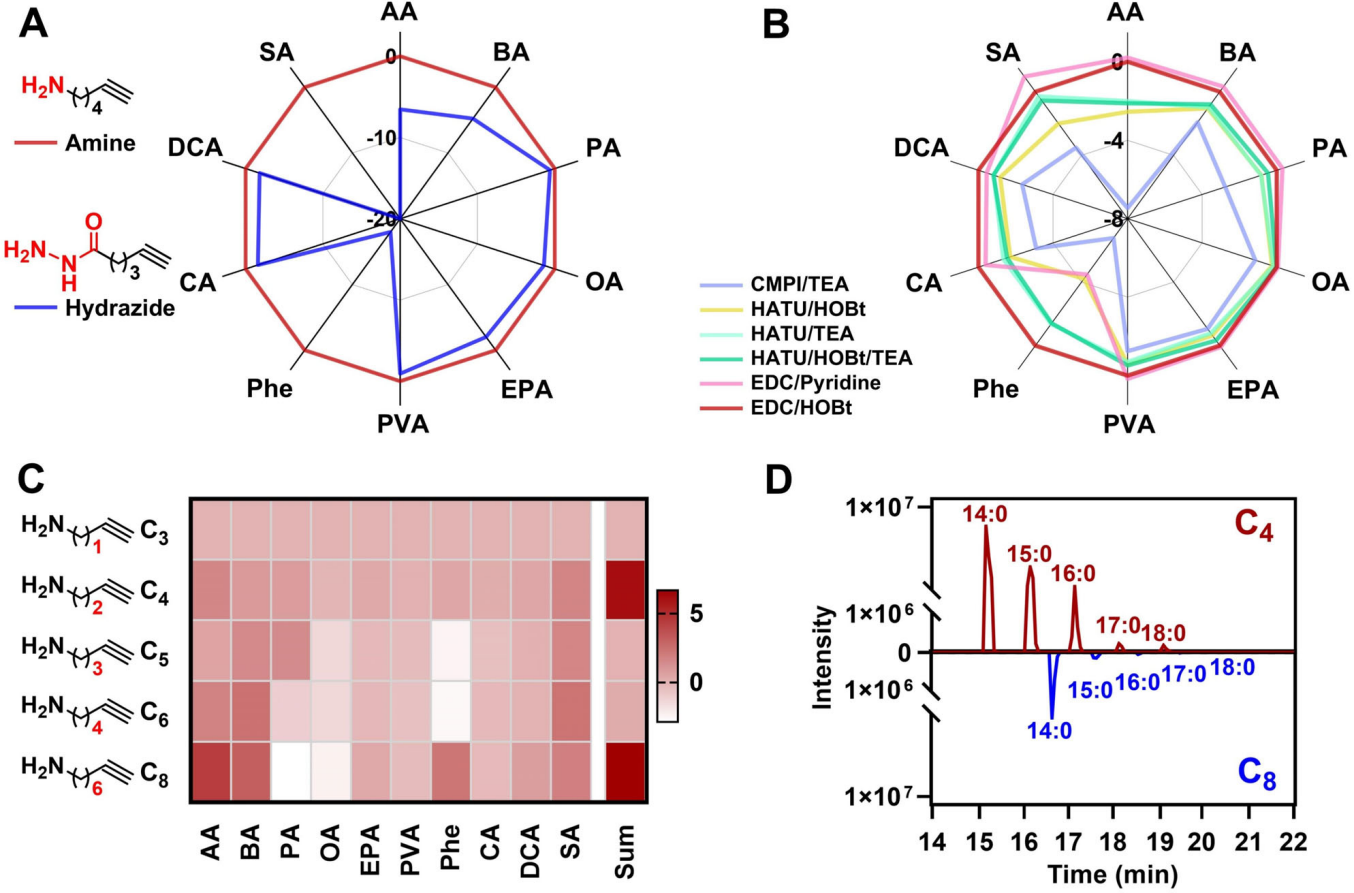
Optimization of probe structure and coupling conditions for carboxyl metabolite encoding. (A) Comparative performance of amine‐ and hydrazide‐based alkyne probes. (B) Effect of condensation reagents on derivatization efficiency. (C) Influence of alkyl chain length (C_3_–C_8_) on labeling efficiency. (D) Comparative MS response of C_4_ and C_8_ probes for representative fatty acids. CMPI, 2‐Chloro‐1‐methylpyridinium iodide. TEA, triethylamine. HATU, *N*‐[(Dimethylamino)‐1*H*‐1,2,3‐triazolo‐[4,5‐*b*]pyridin‐1‐ylmethylene]‐*N*‐methylmethaneminium hexafluorophosphate *N*‐oxide. HOBt, 1‐Hydroxybenzotriazole. EDC, N‐(3‐Dimethylaminopropyl)‐N’‐ethylcarbodiimide. C_3_, propargylamine. C_4_, but‐3‐ynylamine. C_5_, pent‐4‐yn‐1‐amine. C_6_, hex‐5‐yn‐1‐amine. C_8_, 7‐octyn‐1‐amine.

To maximize encoding efficiency and analytical sensitivity, we systematically optimized probe structure and reaction parameters using carboxyl metabolites as representative substrates. Carboxyl‐containing metabolites are widely distributed across diverse structural classes and commonly require activation for efficient derivatization, making them an appropriate benchmark for evaluating probe performance. A panel of ten structurally distinct carboxyl metabolites (10 µM each), including short‐chain fatty acids (FAs), long‐chain FAs, aromatic acids, bile acids, amino acids, and dicarboxylic acids (Figure S1), was selected to assess probe reactivity, linker length, and coupling chemistry. Comparative evaluation of amine‐ and hydrazide‐functionalized probes with identical linker lengths revealed consistently higher derivatization efficiencies for amine‐based probes (Figure 2A), consistent with their greater nucleophilicity under carbodiimide activation conditions. Condensation reagents were next systematically evaluated under equimolar conditions. Among CMPI/TEA [12], HATU‐based systems [13, 14, 15, 16], and EDC‐based systems [17, 18], the EDC/HOBt combination afforded the highest and most reproducible conversion efficiencies across the metabolite panel (Figure 2B), likely reflecting improved suppression of side reactions and enhanced stabilization of activated intermediates.

We next examined the influence of alkyl linker length in amine‐based probes (C_3_–C_8_). Although the C_4_ and C_8_ variants exhibited comparable overall reactivity (Figure 2C), the C_8_ probe showed reduced performance toward long‐chain FAs, likely due to increased hydrophobic aggregation (Figure 2D). Considering reactivity, solubility, and compatibility across structurally diverse substrates, the C_4_ probe (BYA) was selected as the optimal encoding reagent for carboxyl metabolites. For amine‐containing metabolites, DEB was chosen over propiolic acid (PAc) derivatives because of its superior MS response (Figure S2).

Reaction parameters for both encoding (Step 1) and click‐capture (Step 2) were systematically optimized to maximize chemical conversion, capture efficiency, and quantitative robustness. Optimal encoding conditions were established using 2 mM BYA with EDC/HOBt activation (50 °C, 2 h), affording high conversion efficiencies across all tested substrates. Importantly, key reaction parameters, including probe concentration, temperature, and reaction time, were applied uniformly across all four targeted functional groups. (Figure S3). Click‐capture efficiency was further evaluated by varying solvent composition, resin loading, copper catalyst concentration, ligand (BTTAA), reducing agent, and temperature [7, 19]. The optimal system, consisting of 40% H_2_O / 30% NMP / 30% MeOH with CuSO_4_/BTTAA/ascorbate, achieved near‐quantitative immobilization at 40 °C over 12 h, as evidenced by the high reaction efficiency (Table 1 and Figure S4).

**TABLE 1:** Detailed information on reaction sensitivity and efficiency for the derivatives from 10 carboxyl standards.

				Before MREP (Native)				After MREP (Derivative)					Efficiency (%)		
No	Carboxyl standards	Abbr.	Type	Formula	M.W.	RT (min)	LOD (nM)	Formula	Observed *m/z*	RT (min)	LOD (pM)	Change of LOD	Acylation Reaction	Click Chemistry	Cleavage Reaction
**1**	Acetic acid	AA	SFA, SCFA	C_2_H_4_O_2_	60.0211	—	—	C_11_H_19_N_5_O_2_	254.1604	4.13	6	—	/	98.06	99.10
**2**	Butyric acid	BA	SFA, SCFA	C_4_H_8_O_2_	88.0524	—	—	C_13_H_23_N_5_O_2_	282.1905	5.37	25	—	/	99.04	98.93
**3**	Palmitic acid	PA	SFA, LCFA	C_16_H_32_O_2_	256.2402	21.11	250	C_25_H_47_N_5_O_2_	450.3760	17.22	60	4 167	83.84	99.62	97.61
**4**	Oleic acid	OA	MUFA	C_18_H_34_O_2_	282.2559	21.42	100	C_27_H_49_N_5_O_2_	476.3988	17.76	80	1 250	97.78	99.98	99.58
**5**	Eicosapentaenoic acid	EPA	PUFA	C_20_H_30_O_2_	302.2246	18.95	7.5	C_29_H_49_N_5_O_2_	496.3615	15.68	5	1 500	98.67	>99.99	99.61
**6**	5‐Phenylvaleric acid	PVA	Aromatic acid	C_11_H_14_O_2_	178.0994	11.59	1 000	C_20_H_29_N_5_O_2_	372.2414	9.25	25	40 000	95.41	>99.99	98.25
**7**	Phenylalanine	Phe	Amino acid	C_9_H_11_NO_2_	165.0790	3.57	2 ‐500	C_18_H_26_N_6_O_2_	359.2207	5.42	250	10 000	98.66	99.80	99.79
**8**	Cholic acid	CA	Bile acid	C_24_H_40_O_5_	408.2876	12.42	0.5	C_33_H_55_N_5_O_5_	602.4280	10.74	10	50	99.97	99.99	99.70
**9**	Deoxycholic acid	DCA	Bile acid	C_24_H_40_O_4_	392.2927	14.68	2.5	C_33_H_55_N_5_O_4_	586.4344	12.21	75	33	99.92	>99.99	99.78
**10**	Succinic acid	SA	TCA	C_4_H_6_O_4_	118.0266	—	—	C_22_H_36_N_10_O_4_	505.2989	5.01	50	—	/	96.42	99.81

Abbr., abbreviation; RT, retention time; ‐, undetectable; LOD, limit of detection; SFA, saturated fatty acid; SCFA, short‐chain fatty acid; LCFA, long‐chain fatty acid; MUFA, monounsaturated fatty acid; PUFA, polyunsaturated fatty acid.  Reaction efficiencies were calculated using peak intensities before and after reactions.

Cleavage conditions were optimized using TFA/DCM (95:5, *v*/*v*), yielding efficient release of immobilized derivatives without detectable degradation (Figure S5A–C). The composition of the reconstitution solvent significantly influenced MS response: acetonitrile enhanced detection of long‐chain FAs, whereas aqueous media favored short‐chain species. A 50% aqueous acetonitrile solution provided balanced signal intensities across structurally diverse metabolite classes (Figure S5D). MS acquisition parameters were further optimized to ensure sensitive and reproducible detection of the derivatized metabolites (Figure S6).

Under the optimized conditions, the platform achieved high encoding efficiency (>83.8%), capture efficiency (>96.0%), and cleavage efficiency (>88.3%) across all tested metabolites (Table 1 and Table S1 and Figures S7 and S8), demonstrating robust chemical conversion and reproducibility.

### 2.3 Improved Sensitivity and MS Characteristics upon MREP

**FIGURE 3:**
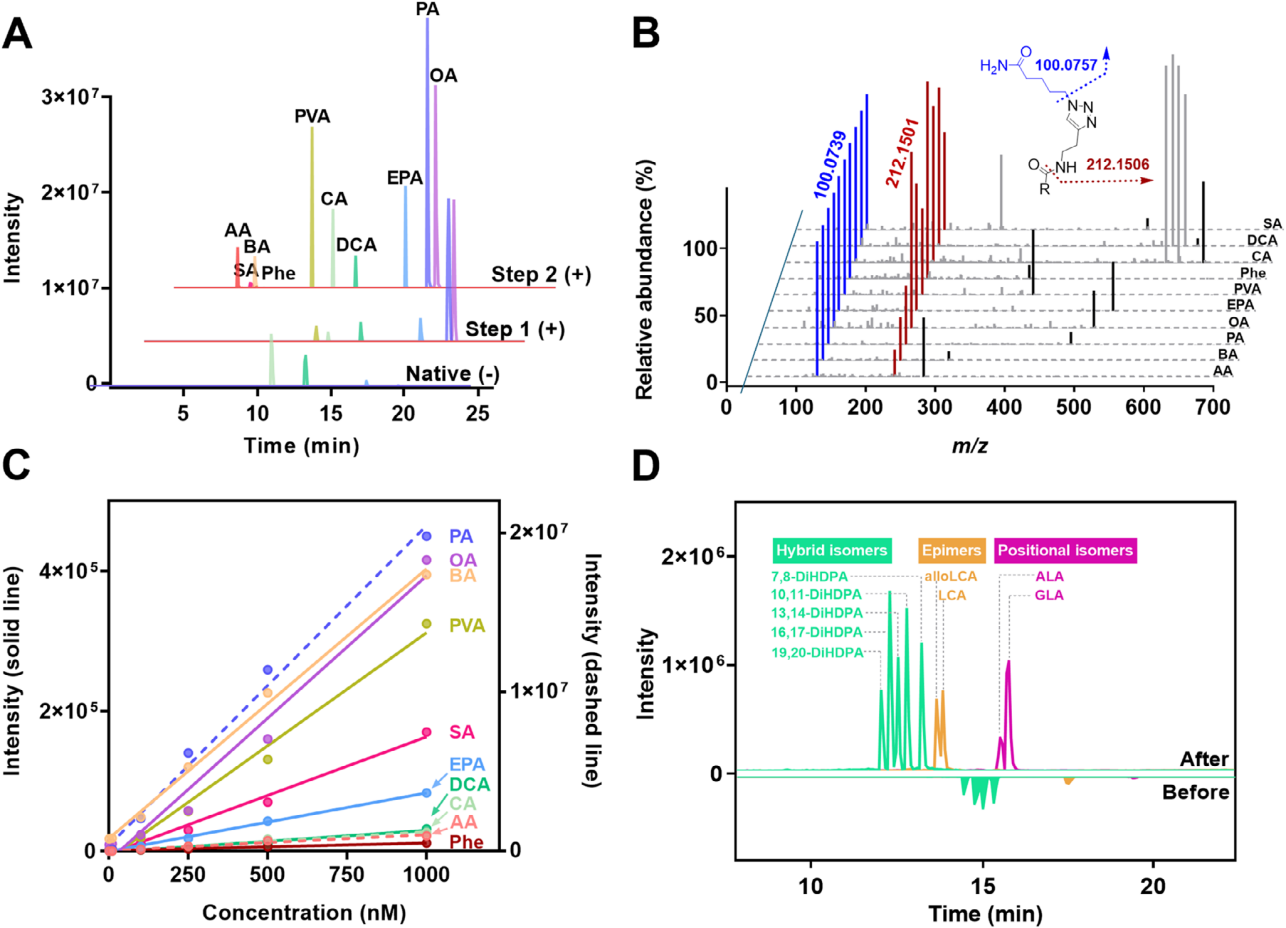
Analytical performance of MREP using representative carboxyl standards. (A) Extracted ion chromatograms (EICs) of ten representative carboxyl metabolites before encoding (detected in negative ion mode), after Step 1 derivatization, and following solid‐phase capture (detected in positive ion mode). (B) MS/MS spectrum of the representative encoded derivatives showing the diagnostic reporter‐ion (blue, *m/z* 100.0739) and a characteristic fragment ion (red, *m/z* 212.1501). (C) Calibration curves demonstrating quantitative linearity following MREP treatment. (D) Improved chromatographic resolution and detection sensitivity for representative carboxyl isomers following MREP.

Using the optimized encoding and capture conditions, a representative panel of carboxyl metabolites was analyzed by LC‐MS before and after MREP treatment (Figure 3A). In their native forms, several short‐chain FAs, including acetic acid (AA), butyric acid (BA), and succinic acid (SA), were undetectable even in negative ion mode, likely owing to intrinsically low ionization efficiencies and poor retention on the reversed‐phase columns. Other metabolites, including phenylalanine (Phe), 5‐phenylvaleric acid (PVA), palmitic acid (PA), oleic acid (OA), and eicosapentaenoic acid (EPA), produced only weak signals. Following MREP, all ten carboxyl metabolites were readily detected as their derivatized forms in positive ion mode. Signal intensities increased markedly after the encoding step and were further amplified upon solid‐phase capture. This pattern highlights the synergistic contribution of chemical transformation and matrix removal, supporting the necessity of the two‐step workflow. Across the metabolite panel, including carboxyl, amine, carbonyl, and thiol derivatives, limits of detection (LOD) improved by 20‐ to 40 000‐fold relative to native LC‐MS analysis (Table 1 and Table S1). This pronounced sensitivity enhancement is attributed to improved ionization efficiency and chromatographic retention following derivatization, particularly for hydrophilic metabolites that otherwise exhibit early elution and limited MS response. MS/MS analysis revealed highly reproducible fragmentation behavior. Carboxyl derivatives exhibited a dominant diagnostic reporter‐ion at *m/z* 100.0757 ([C_5_H_10_NO]^+^) and a characteristic fragment at *m/z *212.1506 ([C_9_H_18_N_5_O]^+^) (Figure 3B). Notably, the *m/z* 100.0757 reporter‐ion was consistently observed across amine, carbonyl, and thiol derivatives, establishing its universal diagnostic value within the platform. Additional class‐specific fragments further supported functional‐group assignment, including *m/z* 237.1352 ([C_11_H_17_N_4_O_2_]^+^) for carbonyl derivatives, and a neutral loss of 45.0215 Da (CH_3_NO) for amine derivatives (Figure S9). These conserved fragmentation signatures enable confident submetabolome classification and facilitate automated metabolite annotation.

Quantitative evaluation demonstrated excellent linearity between signal intensity and metabolite concentration over a 1–1 000 nM range (R^2^ > 0.961) (Figure 3C). Accurate quantification was further validated using 4‐carboxybenzaldehyde (CBA), a bifunctional metabolite containing both carboxyl and carbonyl groups. Independent encoding with BYA and AHZ yielded linear responses (R^2^ > 0.998) across 1–100 µM (Figure S10), confirming quantitative robustness across distinct reactivity classes. Consistent quantitative trends were also observed for cysteine in liver and serum samples across amine‐ and thiol‐targeting workflows (Figure S11). The platform further exhibited high precision, stability, and reproducibility (Table S2). Beyond sensitivity enhancement, MREP substantially improved chromatographic performance, enabling clear resolution of positional, epimeric, and hybrid isomers that were poorly separated in their native forms (Figure 3D and Figure S12). Collectively, these results indicate that MREP not only enhances MS response but also introduces structural regularization that improves fragmentation consistency and chromatographic discrimination.

### 2.4 High Chemoselectivity and Enrichment Performance

**FIGURE 4:**
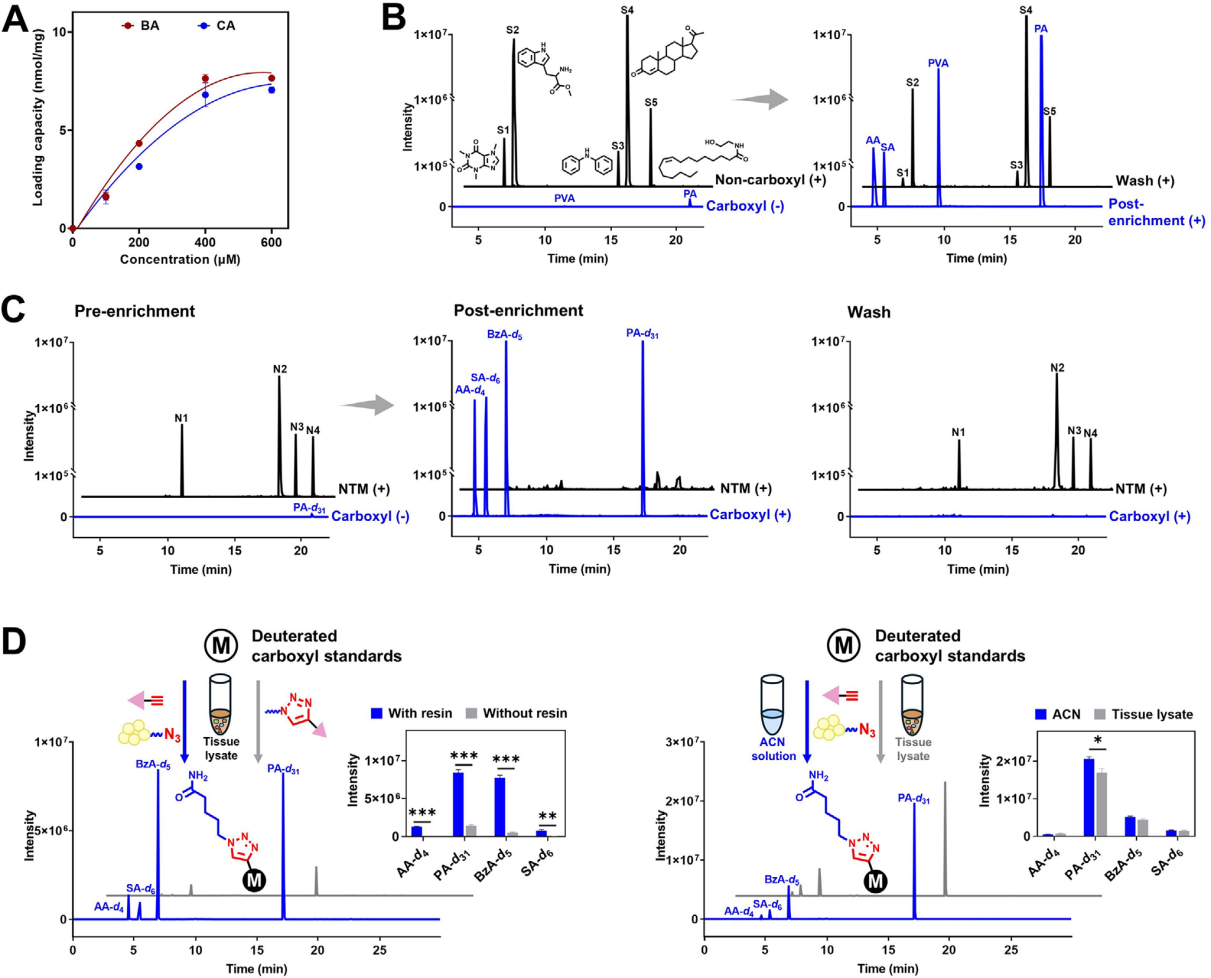
Chemoselectivity and enrichment performance of MREP. (A) Loading capacity of ACER resin evaluated using butyric acid (BA) and cholic acid (CA). (B) Extracted ion chromatograms (EICs) from chemoselectivity assessment using mixed standards. (C) Chemoselectivity validation in liver lysate spiked with deuterated carboxyl standards. Non‐target metabolites (NTM) detected prior to enrichment were absent from the enriched fractions but present in wash eluates. (D) Evaluation of matrix effects using deuterated standards in acetonitrile and liver lysate under different processing conditions (n = 3). Data were expressed as the mean ± SEM, with ^*^ *p* < 0.05, ^**^ *p* < 0.01, ^***^ *p* < 0.001, and p values were from Student's *t*‐test.

To evaluate chemoselectivity and enrichment efficiency, carboxyl metabolites were used as representative substrates. The azide‐functionalized ACER resin exhibited a loading capacity of 7.9 nmol mg^−1^ (Figure 4A), approximately tenfold higher than that of a previously reported chemoselective capture system [8], indicating substantial enrichment capability. Batch‐to‐batch variation and long‐term storage stability of the ACER resin were also assessed and demonstrated consistent performance (Figure S13). Under optimized conditions, the BYA probe displayed exclusive reactivity toward carboxyl groups. Chemoselectivity was initially assessed using a synthetic mixture containing four carboxyl acids (AA, SA, PVA, and PA) and five non‐carboxyl compounds (S1–S5) bearing carbonyl, ester, amide, or hydroxyl functionalities. Following reactivity encoding and solid‐phase capture, non‐carboxyl species were detected only in pre‐enrichment or wash fractions, whereas carboxyl derivatives were observed exclusively in the post‐capture eluate (Figure 4B). Selectivity was further validated in complex biological matrices using deuterated carboxyl standards (AA‐*d*_4_, PA‐*d*_31_, BzA‐*d*_5_, and SA‐*d*_6_) spiked into liver lysates. After MREP treatment, deuterated carboxyl metabolites were efficiently recovered in the eluate, while non‐carboxyl metabolites (N1‐N4) remained confined to wash fractions (Figure 4C). These results confirm the high chemoselectivity and enrichment specificity of the platform in biologically relevant environments.

The solid‐phase capture step further reduces matrix interference by physically separating encoded metabolites from endogenous background species. Comparative analysis of deuterated standards processed with and without resin enrichment revealed markedly higher signal intensities for the captured derivatives. Comparable responses observed in acetonitrile and liver lysate matrices indicate minimal matrix effects (Figure 4D). Consistent with these observations, comparison of one‐step derivatization and two‐step MREP workflowdemonstrated that probe‐encoded derivatization followed by solid‐phase capture significantly enhances signal intensity (Figure S14). In addition, this modular design also enables flexible extension to diverse functional groups without requiring the synthesis of probe‐functionalized resins for each target class. Collectively, these findings indicate that integrating chemoselective encoding with solid‐phase capture facilitates efficient enrichment, spectral simplification, and robust detection of defined functional‐group subsets in complex biological samples.

### 2.5 In‐House Reference Library for Confident Annotation

Reliable metabolite annotation is critical for reactivity‐encoded metabolomics, where structural transformation necessitates accurate reference matching. To enable confident identification, we constructed an in‐house reference library comprising 115 derivatized carboxyl standards spanning diverse chemical classes, including fatty acids, eicosanoids, bile acids, amino acids, aromatic acids, and tricarboxylic acid (TCA) cycle intermediates (Table S3). Each standard was individually encoded and analyzed to establish high‐confidence retention time (RT), MS^1^, and MS^2^ signatures. Because chromatographic retention is highly sensitive to gradient composition, column aging, and instrument variability, RT drift represents a major obstacle to cross‐batch annotation [20]. To address this challenge, we implemented a local linear regression‐based calibration algorithm [20, 21]. Nineteen saturated FA standards, selected for their structural homology and uniform distribution across the chromatographic window (Figure S15A), were employed as external calibrants. Across variations in elution gradients, chromatographic columns, and LC systems (Figure S15B–D and Table S4), regression analysis demonstrated excellent linear correlations (R^2^> 0.999). After calibration, RT deviations for test standards were reduced to <5%, establishing a transferable alignment framework for accurate submetabolome annotation under diverse analytical conditions. This calibration strategy enables reliable integration of MREP with large‐scale metabolite identification workflows.

### 2.6 Profiling of Four Functional‐Group‐Defined Submetabolomes in Biological Samples

**FIGURE 5:**
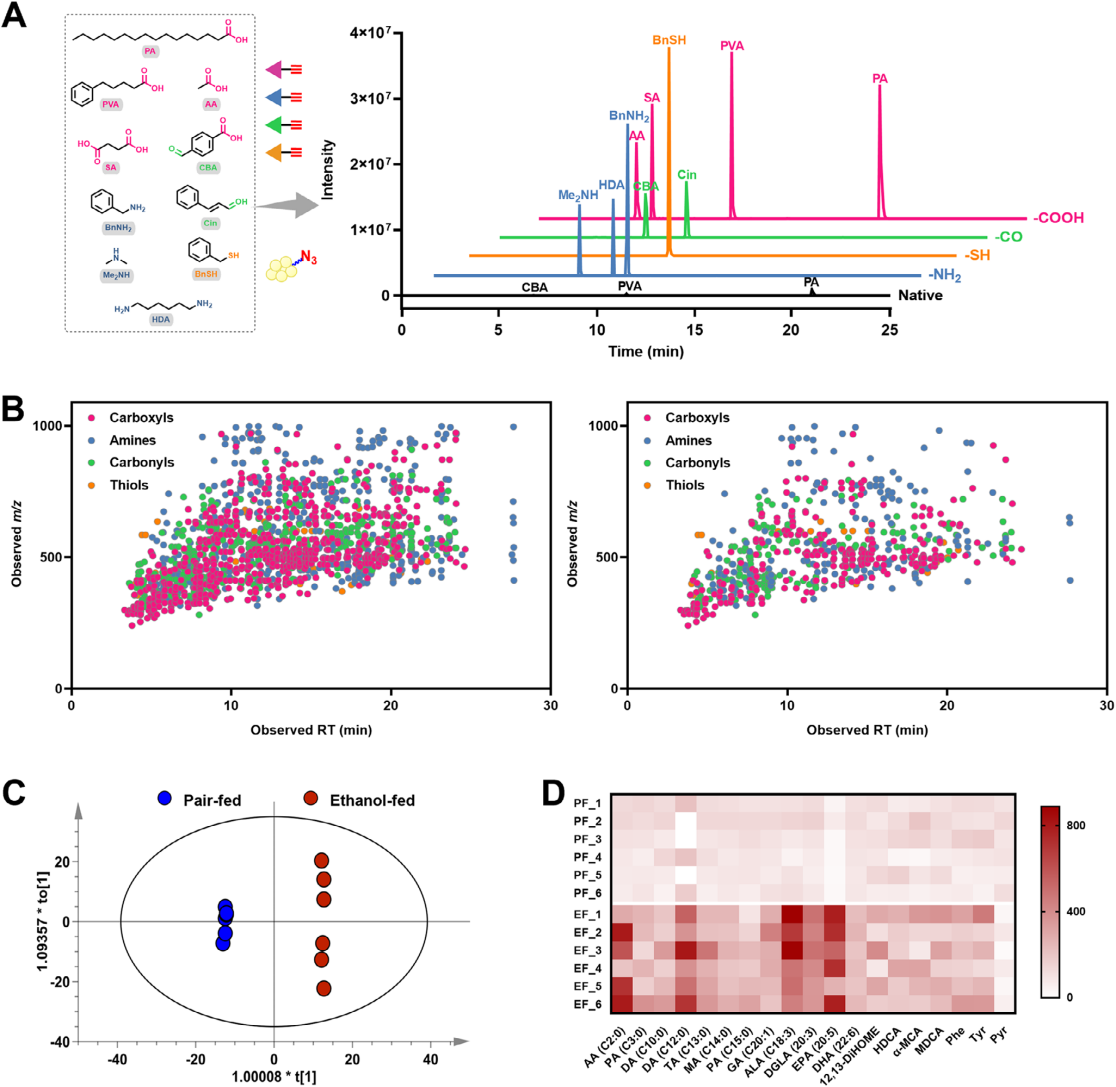
Functional group‐resolved metabolomic profiling. (A) Representative standards from four functional‐group classes (left) and the corresponding extracted ion chromatograms (EICs, right) before and after MREP. (B) Global feature distribution of four functional‐group‐defined metabolite layers in liver (left) and serum (right) samples. Features are plotted by retention time and *m/z* and color‐coded by class. (C) OPLS‐DA score plot of liver submetabolomes from pair‐fed and ethanol‐fed mice. (D) Heatmap of significantly altered carboxyl metabolites between groups.

To evaluate multiplexed reactivity encoding, a mixed standard panel containing representative carboxyls (AA, SA, PVA, and PA), amines (BnNH_2_,  Me_2_NH, and HDA), carbonyls (CBA and Cin), and thiols (BnSH) was subjected to the MREP workflow. Before encoding, most analytes were undetectable under conventional LC‐MS conditions. Following MREP, all metabolites were selectively detected in their respective functional‐group channels in positive ion mode (Figure 5A), confirming orthogonality and channel specificity.

We next applied MREP to profile four functional‐group‐defined submetabolomes in liver tissue and serum samples from mice. Metabolites were extracted, divided into four aliquots, independently encoded, captured on ACER resin, and analyzed by high‐resolution LC‐MS using MS^E^ acquisition to simultaneously record precursor and fragment ions. Feature annotation was guided by the universal reporter‐ion (*m/z* 100.0757) in combination with class‐specific fragmentation signatures, enabling systematic classification of encoded features. Across all samples, 7 208 features were assigned as putative metabolites, of which 1 573 (liver) and 656 (serum) were structurally annotated (Figure 5B and Tables S5–S9). Sixty‐seven metabolites were further validated against the in‐house library through calibrated RT and MS/MS matching, confirming annotation accuracy in complex biological matrices.

Multivariate analysis of liver samples (n  =  6 per group) revealed clear separation between ethanol‐fed and pair‐fed mice (Figure 5C), indicating pronounced metabolic reprogramming across the four reactivity‐defined layers. Among 191 significantly altered metabolites (*p* < 0.05, VIP > 1), 19 were validated by reference standards (Figure 5D). Observed alterations included elevated short‐chain FAs (C2:0  and C3:0) [22, 23], monounsaturated FAs (C20:1) [23] and polyunsaturated FAs (ALA, DGLA, EPA, and DHA) [23, 24], eicosanoids (12,13‐DiHOME) [25], and aromatic amino acids (phenylalanine and tyrosine) [26, 27], accompanied by decreased pyruvic acid level [28]. These trends are consistent with previous reports of alcohol‐induced metabolic remodeling. Notably, several saturated FAs (C10:0–C15:0) and bile acids (HDCA and MDCA) were additionally detected under this analytical framework. Collectively, these results indicate that MREP enables high‐coverage and quantitative stratification of metabolomes across multiple functional classes. The observed metabolic alterations reflect coordinated shifts in lipid and energy metabolism associated with alcohol‐induced metabolic reprogramming, underscoring the biological applicability of the platform for investigating functional metabolic adaptations. By integrating orthogonal chemical transformation, unified solid‐phase capture, and diagnostic fragmentation logic, MREP provides a scalable framework for structurally resolved interrogation of complex biochemical systems.

## 3 Conclusion

In this work, we establish MREP as a strategy for functional group‐resolved stratification of complex metabolomes. By integrating orthogonal chemoselective transformations with a unified solid‐phase capture architecture, the platform converts chemically heterogeneous small molecules into structurally encoded derivatives that share common ionization and fragmentation logic. This reactivity‐guided regularization overcomes intrinsic disparities in ionization efficiency and matrix interference that constrain conventional LC‐MS analyses. The high conversion efficiency, capture fidelity, and consistent reporter‐ion behavior collectively demonstrate that chemical encoding can serve not merely as a sensitivity enhancement tool, but as a structural organizing principle for metabolomic interrogation.

Application to mouse liver and serum samples illustrates the capacity of this framework to expand detectable chemical space and enable confident annotation in biologically complex systems. Across four reactivity‐defined layers, thousands of encoded features were detected, with a substantial subset validated against an in‐house reference library. Differential analysis of ethanol‐fed and pair‐fed mice revealed coordinated perturbations primarily within carboxyl‐containing metabolite classes, including FAs, bile acids, and amino acids. Many of these metabolites are closely associated with gut microbial metabolism, consistent with disruption of the gut‐liver axis in alcohol‐associated liver disease [29, 30]. While the biological trends align with prior reports, the present study demonstrates that reactivity‐defined stratification provides a chemically resolved and complementary perspective of metabolic remodeling.

Beyond this specific disease model, the modular architecture of the platform enables extension to additional functional groups through rational probe design. Such programmability provides opportunities to progressively expand chemical coverage and construct functionally stratified views of metabolic networks. Nevertheless, several limitations warrant consideration. Acidic cleavage conditions may affect particularly labile metabolites, although evaluation under representative TFA‐based cleavage conditions indicated no significant degradation of derivatized carboxyl metabolites. In addition, cross‐reactivity among densely functionalized species may occur under certain contexts, necessitating tailored reaction conditions or further probe optimization.

Overall, this work establishes chemoselective reactivity encoding as a generalizable chemical framework for metabolomics. By integrating functional‐group transformation, unified solid‐phase capture, and diagnostic fragmentation logic, the MREP provides a scalable platform for structurally resolved, high‐definition interrogation of small‐molecule systems across diverse biological contexts.

## 4 Experimental Section

### 4.1 Materials and Reagents

Metabolite standards (Table S3 and Figure S1) were obtained from Cayman Chemical, Nu‐Chek‐Prep, and Sigma–Aldrich, and other commercial sources. NovaPEG Rink Amide resins and 5‐azidopentanoic acid were purchased from Sigma–Aldrich and Cayman Chemical, respectively. The following reagents were used for alkyne‐tagged probes: C_3_ (Energy Chemical), BYA (C_4_, Bide Pharmatech), C_5_ (Stru Chem), C_6_ (Leyan), C_8_ (Aladdin), DEB (Leyan), PAc (Macklin), AHZ (Lumiprobe), NPM (TCI). Condensation reagents, 2‐(1H‐benzotriazole‐1‐yl)‐1,1,3,3‐tetramethyl‐ ammonium tetrafluoroborate (TBTU), TEA, HATU, and N, N‐diisopropylethylamine (DIEA), were acquired from Aladdin. N, N‐Dimethylformamide (DMF), TFA, and pyridine were obtained from Macklin. BTTAA and TIPS were purchased from Energy Chemical. CMPI and EDC were sourced from Sigma–Aldrich, N‐methyl‐2‐pyrrolidone (NMP) from Adamas, and HOBt was obtained from J&K Scientific.

### 4.2 Animals and Treatments

Male C57BL/6J mice were maintained under specific pathogen‐free conditions. Alcoholic Liver Disease (ALD) was induced using the National Institute on Alcohol Abuse and Alcoholism (NIAAA) chronic‐plus‐binge model as described previously [31, 32, 33, 34]. Briefly, after acclimatization to a Lieber‐DeCarli liquid diet (ROPHIC Animal Feed High‐Tech Co., Ltd., Jiangsu, China), mice received either a Lieber‐DeCarli ethanol (5%, *v*/*v*) liquid diet or an isocaloric maltose dextrin control diet for 10 days. On day 11, ethanol‐fed mice were gavaged with ethanol (5 g kg^−1^), and controls received isocaloric maltose dextrin. After 9 h of fasting, the animals were anesthetized, and serum and liver tissues were collected for subsequent analysis. All procedures were approved by the Animal Research Ethics Committee of the University of Macau (UMARE 037‐2024).

### 4.3 Encoding With Alkyne‐Tagged Probes

Four alkyne‐tagged probes were employed to selectively label metabolites from distinct functional‐group submetabolomes. For each reaction, metabolite extract supernatants or standard solutions were mixed with probe‐specific reagents for targeted derivatization. For carboxyl submetabolome labeling, 100 µL of ACN solution (either standard solutions or reconstituted sample extracts) was mixed with 100 µL BYA (2 mM in ACN), 100 µL EDC (20 mM in ACN), 100 µL HOBt (2 mM in ACN), and 100 µL ACN, giving a final volume of 500 µL. For amine metabolites, 100 µL of sample was combined with 100 µL DEB (2 mM in ACN) and 300 µL ACN. For carbonyl metabolites, 100 µL of sample was mixed with 100 µL AHZ (2 mM in 80% methanol) and 300 µL 80% methanol, while for thiol metabolites, 100 µL of sample was combined with 100 µL NPM (2 mM in ACN) and 300 µL ACN, maintaining the same total reaction volume of 500 µL. All reaction mixtures were vortexed thoroughly and incubated at 50 °C for 2 h to complete derivatization. The resulting labeled metabolites were subsequently treated with ACER resins for selective enrichment [18].

### 4.4 Synthesis of ACER Resin

NovaPEG Rink Amide resin (50 mg, 0.0175 mmol; Novabiochem, Sigma–Aldrich) was pre‐swollen in dry DMF and coupled with 5‐azidopentanoic acid (1.5 equiv.), TBTU (3 equiv.), and DIEA (3 equiv.) at room temperature for 10 h. Reaction completion was verified by a Kaiser test. The azide‐functionalized ACER resin was washed thoroughly, dried under nitrogen, suspended in NMP (40 mg mL^−1^), and stored at 4 °C [35].

### 4.5 Click Reaction and Cleavage Conditions

Probe‐labelled metabolites (intermediates) were reacted with 400 µL of a click reaction cocktail consisting of 75 µL methanol, 125 µL ACER resin suspension (40 mg mL^−1^), 40 µL 50 mM CuSO_4_, 40 µL 100 mM BTTAA, and 120 µL 1 M ascorbic acid (in water). The mixture was incubated on a ThermoMixer at 1 000 rpm and 40 °C for 12 h. After centrifugation at 10 000 rpm for 3 min, the metabolite‐bound resins were collected and washed sequentially with DMF/water (1:3, 500 µL), DMF/water (1:1, 500 µL), and DMF (500 µL), then dried under nitrogen. Bound metabolites were subsequently cleaved from the resin using TFA/DCM (95:5, *v/v*) (300 µL) with agitation at 1 000 rpm and 30 °C for 1 h. The supernatant was collected, evaporated under nitrogen, and the dried residues were re‐dissolved in 100 µL of 50% ACN. Finally, solutions were centrifuged at 14 800 rpm for 10 min, and the supernatants were collected for LC‐MS analysis [7, 35].

### 4.6 Resin Loading Capacity Analysis

Under optimized conditions, two carboxyl standards, including butyric acid (BA) and cholic acid (CA), were used to evaluate the binding capacity of the ACER resin (5 mg). Increasing concentrations of BA and CA (up to 60 nmol) were reacted with the resin. Signal intensities showed a linear correlation between analyte concentration and the amount of capture, followed by a plateau, indicating the maximum labeling capacity of the ACER resin [8, 11].

### 4.7 Reaction Efficiency Evaluation

A panel of ten carboxyl acids (100 µL, 10 µM each) was sequentially reacted with BYA (Step 1, generating intermediates) and ACER resins (Step 2, generating derivatives) under optimized conditions. Reaction efficiencies were determined by comparing LC‐MS peak intensities before and after each reaction [11, 36]. Acylation efficiency (Step 1) was evaluated by the remaining unreacted carboxyl acids in negative ion mode. Click reaction efficiency (Step 2) was calculated from residual intermediates in positive ion mode. Cleavage efficiency was derived from residual derivatives in positive ion mode.

### 4.8 Chemoselectivity Evaluation in Solution and Biological Matrix

The chemoselectivity of the derivatization and enrichment workflow was evaluated in both standard mixtures and biological matrices. A standard mixture containing four carboxyl metabolites (AA, PA, PVA, and SA) and five non‐carboxyl controls (S1‐S5) was prepared in acetonitrile and treated with BYA, followed by reaction with ACER resins. Post‐enrichment solutions (containing derivatized carboxyl metabolites) and washing eluates were analyzed by LC‐MS [7, 11]. For biological validation, a liver tissue lysate was spiked with four deuterated carboxyl standards (AA‐*d*_4_, PA‐*d*_31_, BzA‐*d*_5_, and SA‐*d*_6_), subjected to sequential treatment with BYA and ACER resins. Both post‐enrichment solution and washing fractions were analyzed by LC‐MS [7].

The enrichment performance of ACER resins was further assessed using four deuterated carboxyl standards (AA‐*d*_4_, PA‐*d*_31_, BzA‐*d*_5_, and SA‐*d*_6_). Each standard (10 µM) was spiked into liver tissue lysate and subjected to either (*i*) direct labeling with BYA followed by reaction with ACER resins, or (*ii*) labeling using acid‐cleavage products released from the resins. Parallel tests were also performed by spiking the same deuterated standards into either ACN or liver lysate, followed by identical sequential labeling and enrichment steps [7, 37]. Comparative analysis of LC‐MS signal intensities confirmed high, reproducible enrichment performance of the MREP workflow under optimized conditions.

### 4.9 Sample Preparation and LC‐MS Analysis

For serum samples, 120 µL of serum sample was divided into four aliquots (30 µL each) for the analysis of different functional groups. Metabolites were extracted by protein precipitation using three volumes of ice‐cold methanol [4]. For liver samples, approximately 50 mg of tissue from the same lobe was homogenized in 500 µL of methanol, followed by centrifugation at 12 000 rpm for 10 min at 4 °C. The resulting supernatants were collected for subsequent derivatization and LC‐MS analysis. LC‐MS analysis was performed on a Waters ACQUITY UPLC system coupled to a SYNAPT G2 Si Q‐TOF MS (Waters, UK). Chromatographic separation was achieved using an HSS T3 column (100 × 2.1 mm, 1.8 µm) maintained at 30 °C. Mobile phases consisted of 0.1% formic acid in water (A) and ACN (B), delivered at a flow rate of 0.5 mL min^−1^. The gradient elution program was programmed as follows: 1% B to 100% B over 23 min, held at 100% B for 4 min, then re‐equilibrated at 1% B. The injection volume was 3 µL, and the autosampler temperature was maintained at 10 °C. Mass spectrometric detection of derivatized metabolites was carried out in positive electrospray ionization (ESI^+^) mode with the following optimized parameters: capillary voltage, 2.4 kV; cone voltage, 50 V; sample cone voltage, 30 V; source temperature, 120 °C; desolvation temperature, 550 °C; desolvation gas flow, 700 L h^−1^; cone gas flow, 50 L h^−1^. For native analytes, comparable LC‐MS conditions were applied, except that carboxyl metabolites were analyzed in negative ion mode (ESI^−^), while amine, carbonyl, and thiol metabolites were primarily detected in positive ion mode (ESI^+^), according to their dominant functional groups. Spectra were acquired in MS^E^ mode (*m/z* 50‐1000) with a scan time of 1.0 *s*, and a collision energy ramp of 5–30 V. Leucine‐enkephalin was continuously infused as a lock mass calibrant to ensure mass accuracy throughout the analysis.

### 4.10 Data Processing

Raw LC‐MS data were processed using UNIFI software (Waters, UK). Features containing reporter‐ion (*m/z* 100.0757, corresponding to [C_5_H_10_NO]^+^) in MS/MS spectra were identified using the UNIFI Discovery module, in combination with characteristic fragmentation patterns specific to different functional groups, including diagnostic fragment ions and specific neutral loss. Control samples were processed using the same workflow to exclude background‐derived features. In addition, for each submetabolome, precursor ions were filtered by applying minimal *m/z* thresholds corresponding to derivatized forms: carboxyl metabolites (*m/z* > 240.1455, formic acid derivatives), amine metabolites (*m/z* > 302.1612, methylamine derivatives), carbonyl metabolites (*m/z* > 281.1721, methanal derivatives), and thiol metabolites (*m/z* > 326.1282, methanethiol derivatives). Accurate masses of native metabolites were reconstructed by subtracting the corresponding tag masses: carboxyls, *m/z* 194.1400 ([C_9_H_16_N_5_]^+^); amines, *m/z* 271.1190 ([C_14_H_15_O_2_N_4_]^+^); carbonyls,  *m/z* 251.1615 ([C_11_H_19_ON_6_]^+^); and thiols, *m/z* 278.1248 ([C_12_H_16_O_3_N_5_]^+^). Metabolite annotation was performed using an in‐house library constructed from chemically labeled authentic standards with calibrated RTs, or by matching accurate masses in the Human Metabolome Database (HMDB) within a ± 20 ppm tolerance.

### 4.11 Statistical Analysis

Metabolic differences between ethanol‐fed and pair‐fed mice were evaluated using OPLS‐DA implemented in SIMCA‐P (Version 14.0, Umetrics, Umeå, Sweden). Quantitative data are presented as mean ± SEM, and statistical significance was determined using Student's *t*‐test, with *p* < 0.05 considered statistically significant. Bar plots were generated using GraphPad Prism 8.0 (GraphPad Software, La Jolla, CA, USA).

## Author Contributions

J.W. designed the study. X.T. performed the experiment, analyzed the data, and drafted the initial manuscript. C.Z., R.Y., M.R., Z.Z., J.L., J.Z., K.W., and R.H. contributed to the experiment. J.W. critically reviewed and revised the manuscript. J.W. provided funding. J.W. and H.L. supervised the project.

## Funding

The work was financially supported by grants from the University of Macau and University of Macau Development Foundation (MYRG‐GRG2024‐00097‐ICMS‐UMDF), and the Science and Technology Development Fund, Macau SAR (File no. 0070/2025/AFJ, 0002/2025/NRP, and 0008/2025/EQP).

## Conflicts of Interest

The authors declare no conflicts of interest.

## Supporting information




**Supporting File 1**: advs76599‐sup‐0001‐SuppMat.docx.

**Supporting File 2**: advs76599‐sup‐0002‐TablesS1‐S9.xlsx.

## Data Availability

The data that support the findings of this study are available from the corresponding author upon reasonable request.
