# Supplementary material for: Chemoselective Metabolomics via a Modular Reactivity‐Encoding Platform
Source: Adv Sci (Weinh). 2026 Jul 17:e76599. Online ahead of print. doi: 10.1002/advs.76599 (PMC13379249; doi:10.1002/advs.76599)
Supplement: Supplementary file 1 — Supporting File 1: advs76599‐sup‐0001‐SuppMat.docx. [file ADVS-9999-e76599-s001.docx]

Supporting Information

**Chemoselective Metabolomics via a Modular Reactivity-Encoding Platform**

Xin Tao^1^, Cang-Man Zhang^1^, Ru-Jie Yang^1^, Min Ren^1^, Zhuo-Ling Zhong^1^, Kang-Hui Wang^1^, Rui-Xue Hu^1^, Jia-Yue Liu^1^, Jun-Yi Zhou^1^, Hou-Kai Li^2^, Jian-Bo Wan^1,*^

**Table of contents**

Figure S1. Chemical structures of standards S1

Figure S2. Comparison of different amine-reactive probes S2

Figure S3. Optimization of Step 1 reaction conditions S3

Figure S4. Optimization of Step 2 reaction conditions S4

Figure S5. Optimization of cleavage conditions S5

Figure S6. Optimization of detection conditions S6

Figure S7. Workflow for calculating reaction efficiency S7

Figure S8. EICs of standards before and after derivatization S8

Figure S9. EICs and MS/MS spectra of standards S9

Figure S10. EICs of CBA derivatives S10

Figure S11. EICs of cysteine derivatives S11

Figure S12. EICs of representative carboxyl isomers S12

Figure S13. Evaluation of variation and stability of the resin S13

Figure S14. Comparison of one-step and two-step strategies S14

Figure S15. RT calibration S15

**
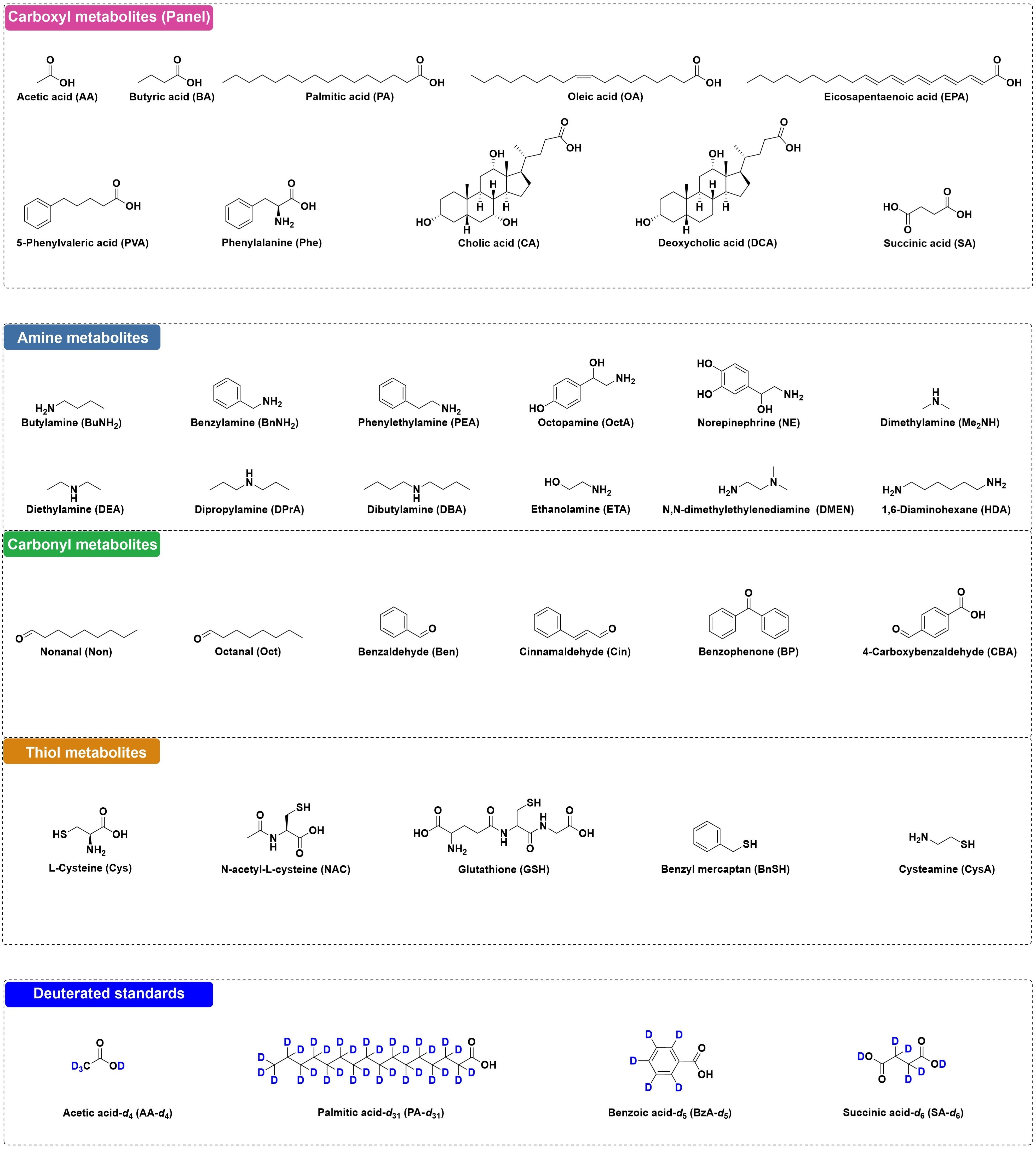
Figure S1.** Chemical structures of representative carboxyl, amine, carbonyl, and thiol standards used in this study.


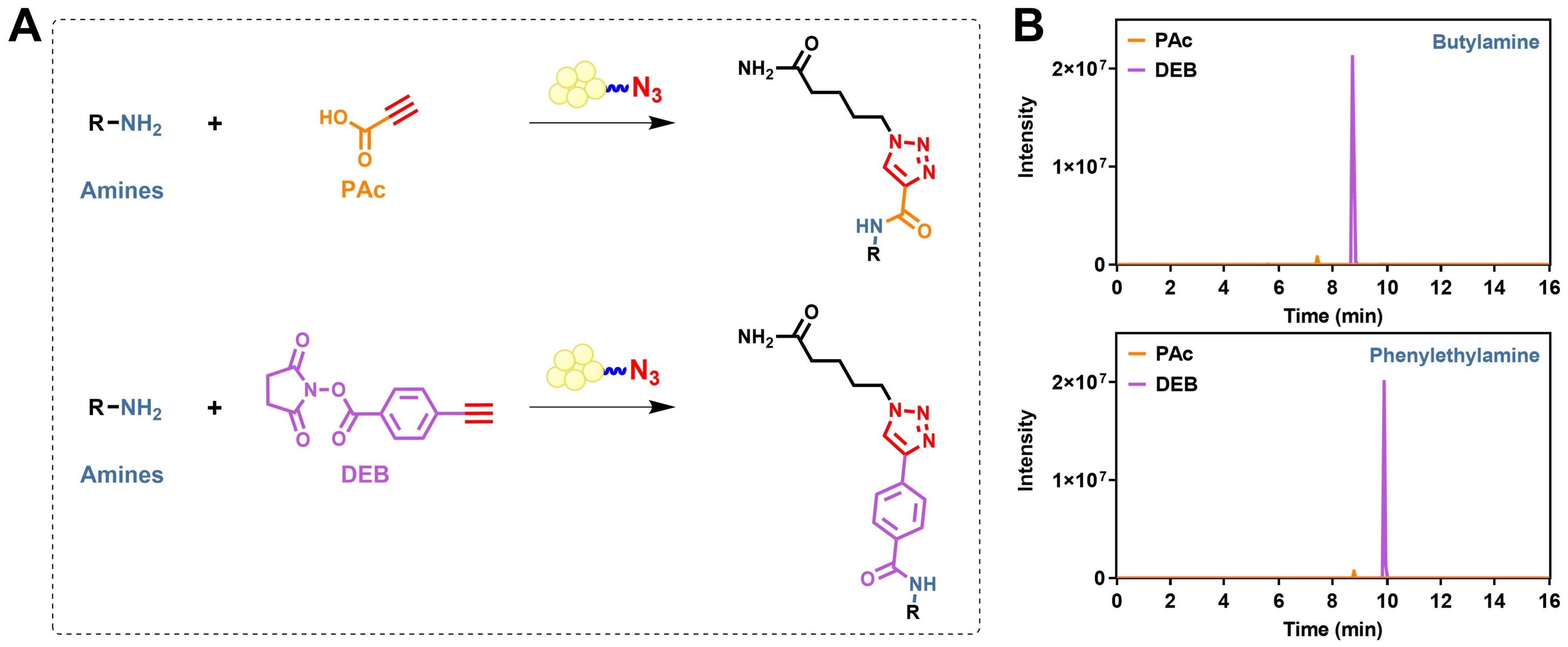


**Figure S2.** Optimization of alkyne-tagged probes for amine metabolite derivatization. (A) Comparison of amine‑reactive probes, propiolic acid (PAc) and 2,5‑dioxopyrrolidin‑1‑yl 4‑ethynylbenzoate (DEB). (B) Extracted ion chromatograms (EICs) of representative amine metabolites, butylamine and phenylethylamine, derivatized with PAc and DEB.


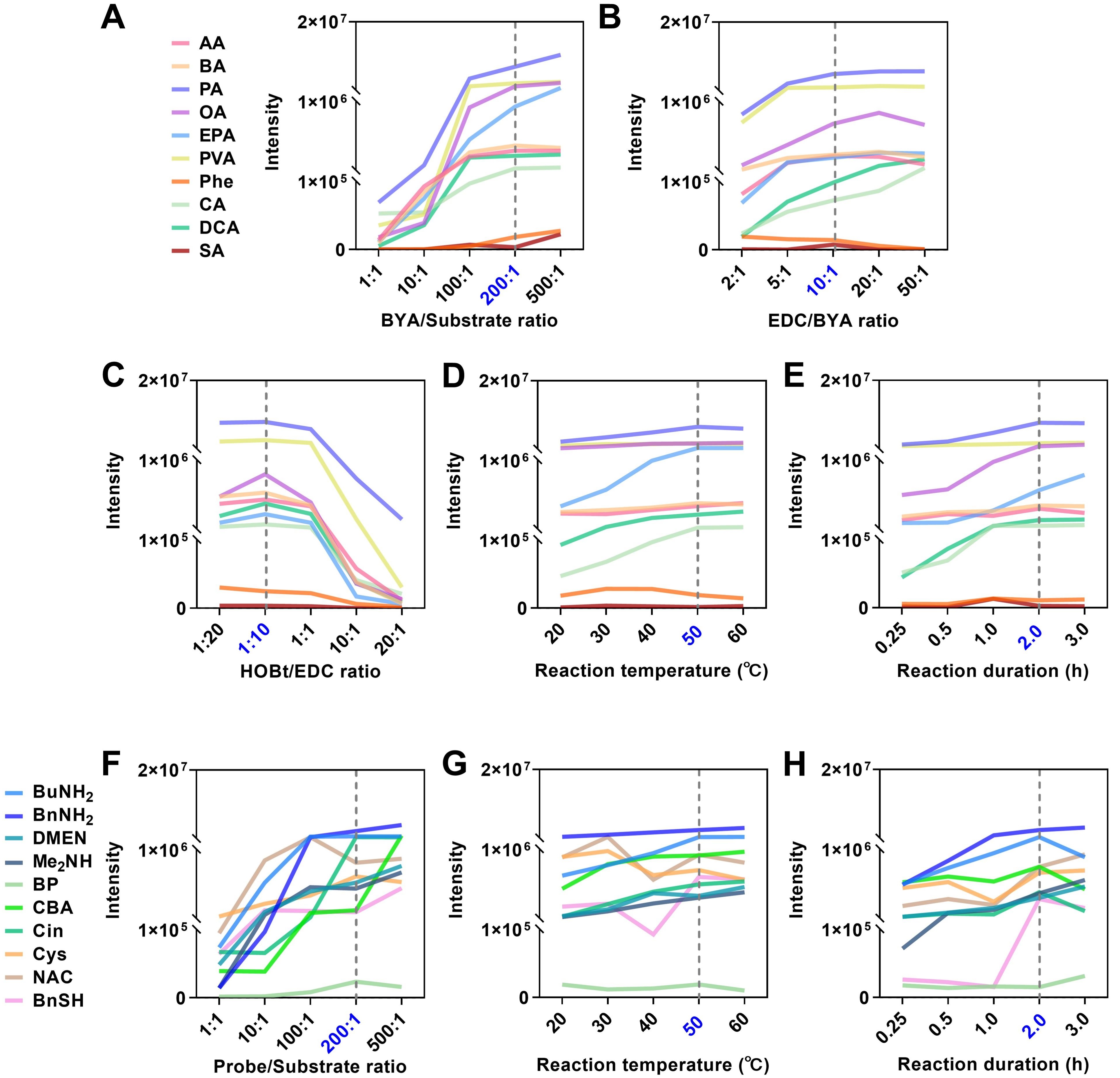


**Figure S3.** Optimization of Step 1 derivatization conditions for carboxyl, amine, carbonyl and thiol metabolites using twenty representative standards (ten carboxyl standards, four amine standards, three carbonyl standards and three thiol standards). Effects of (A) BYA concentration, (B) EDC concentration, (C) HOBt concentration, (D) reaction temperature, (E) reaction time for carboxyl metabolites, (F) probe concentration, (G) reaction temperature, and (H) reaction time for amine, carbonyl and thiol metabolites.


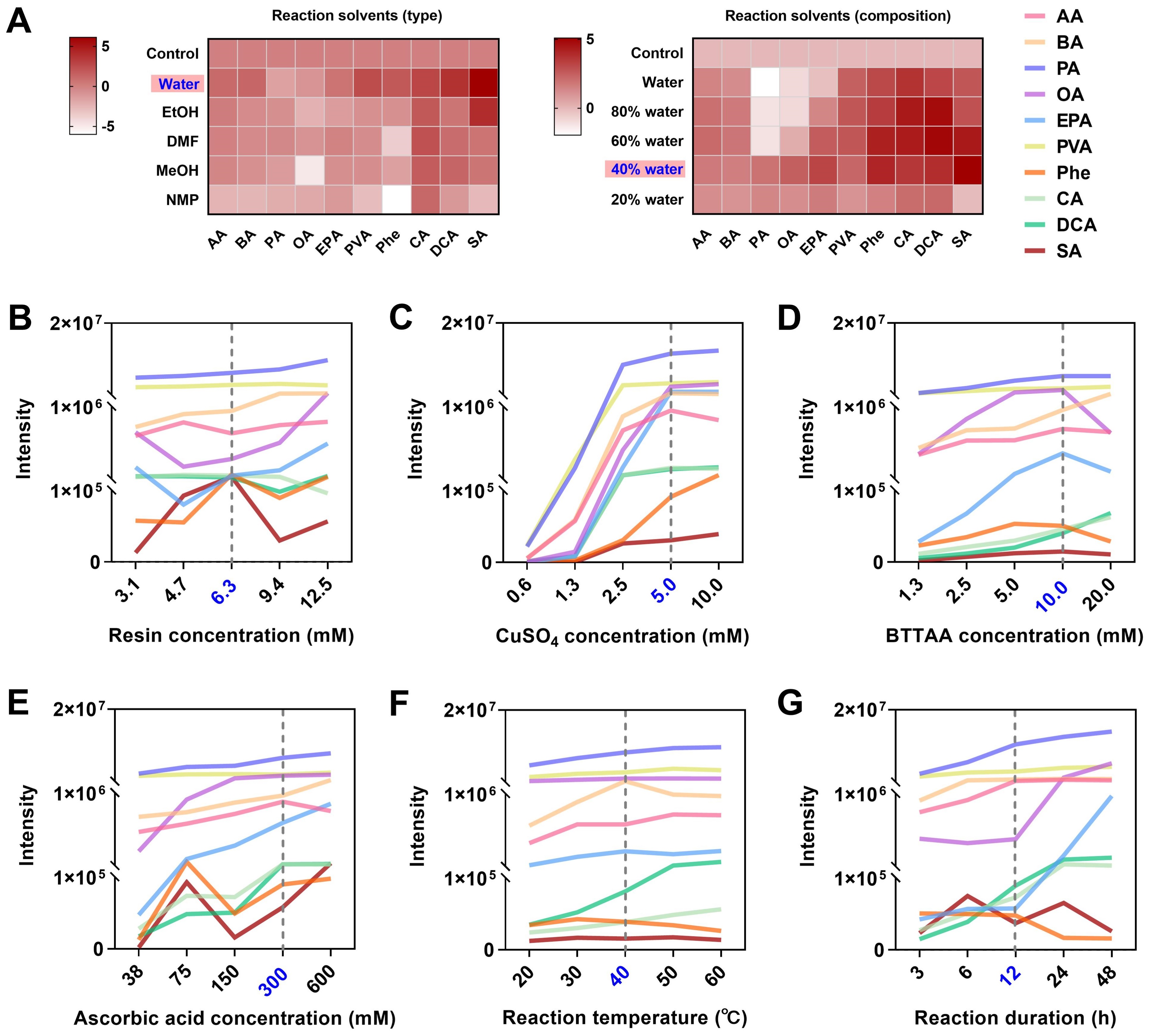


**Figure S4.** Optimization of click-reaction conditions (Step 2) for carboxyl metabolites using ten representative standards. Effects of (A) reaction solvent type (left) and **composition (right)**, (B) resin amount, (C) CuSO₄ concentration, (D) BTTAA concentration, (E) ascorbic acid concentration, (F) reaction temperature, and (G) reaction time. BTTAA, 2-(4-((bis((1-(tert-butyl)-1H-1,2,3-triazol-4-yl)methyl) amino)methyl)-1H-1,2,3-triazol-1-yl) acetic acid.


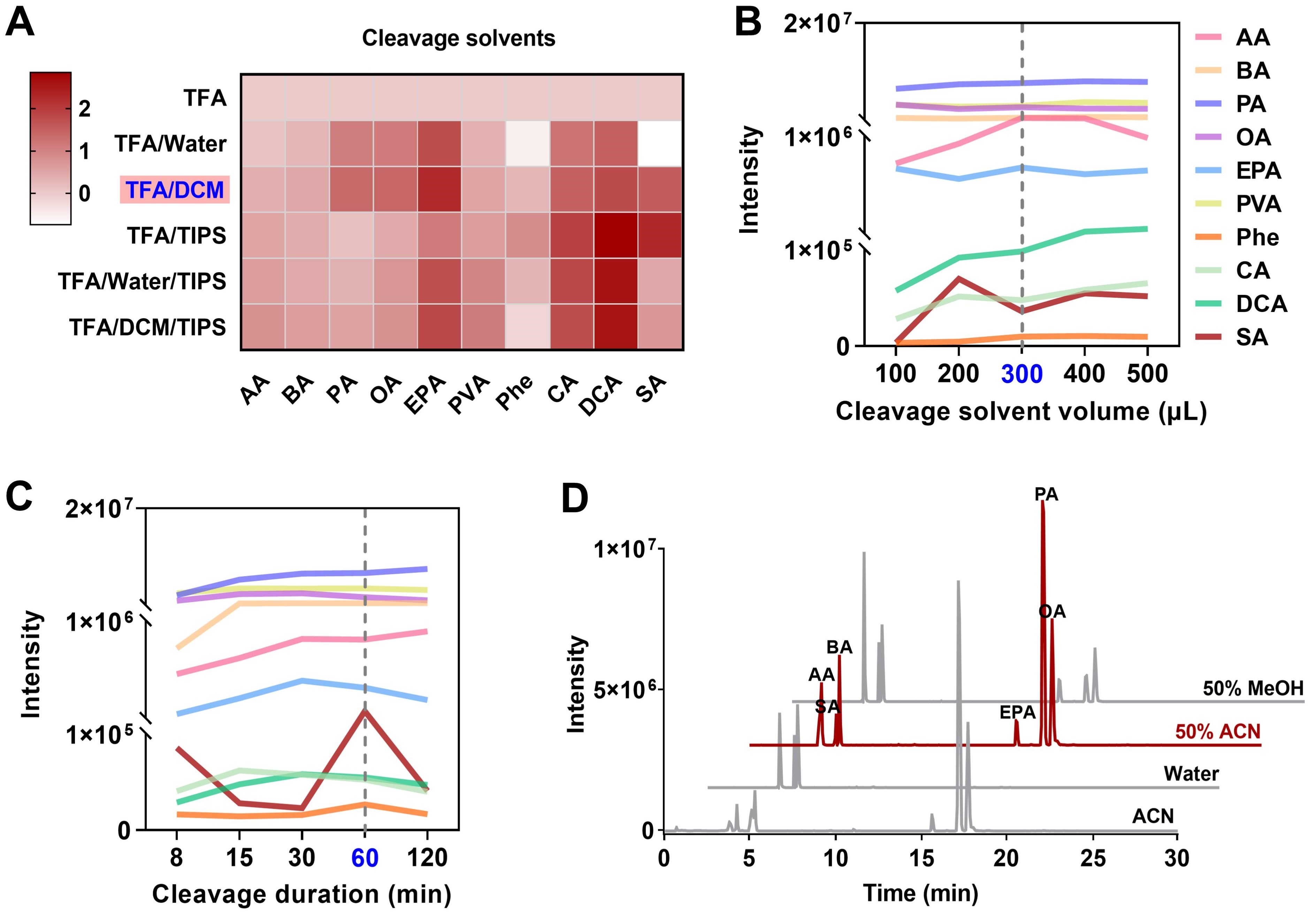


**Figure S5.** Optimization of cleavage conditions for carboxyl metabolites. Effects of (A) cleavage solvent **type**, (B) cleavage solvent volume, (C) cleavage time, and (D) reconstitution solvents. The abbreviations of carboxyl standards correspond to those shown in Figure S1. TFA, trifluoroacetic acid. DCM, dichloromethane. TIPS, triisopropylsilane.


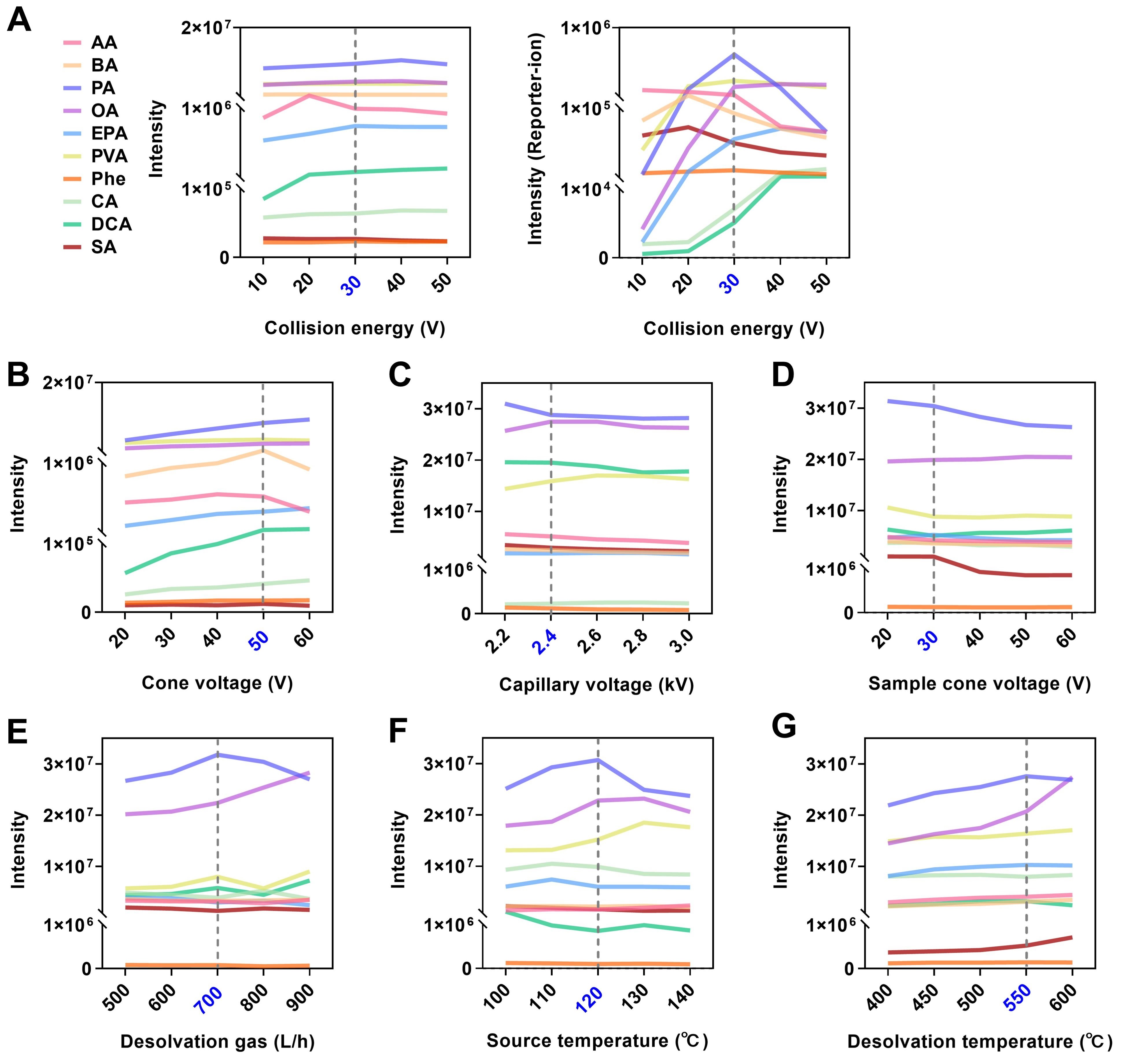


**Figure S6.** Optimization of MS parameters for encoded carboxyl metabolites using ten representative standards, including (A) collision energy (left, MS^1^; right, reporter-ion in MS^2^), (B) cone voltage, (C) capillary voltage, (D) sample cone voltage, (E) desolvation gas, (F) source temperature, and (G) desolvation temperature.


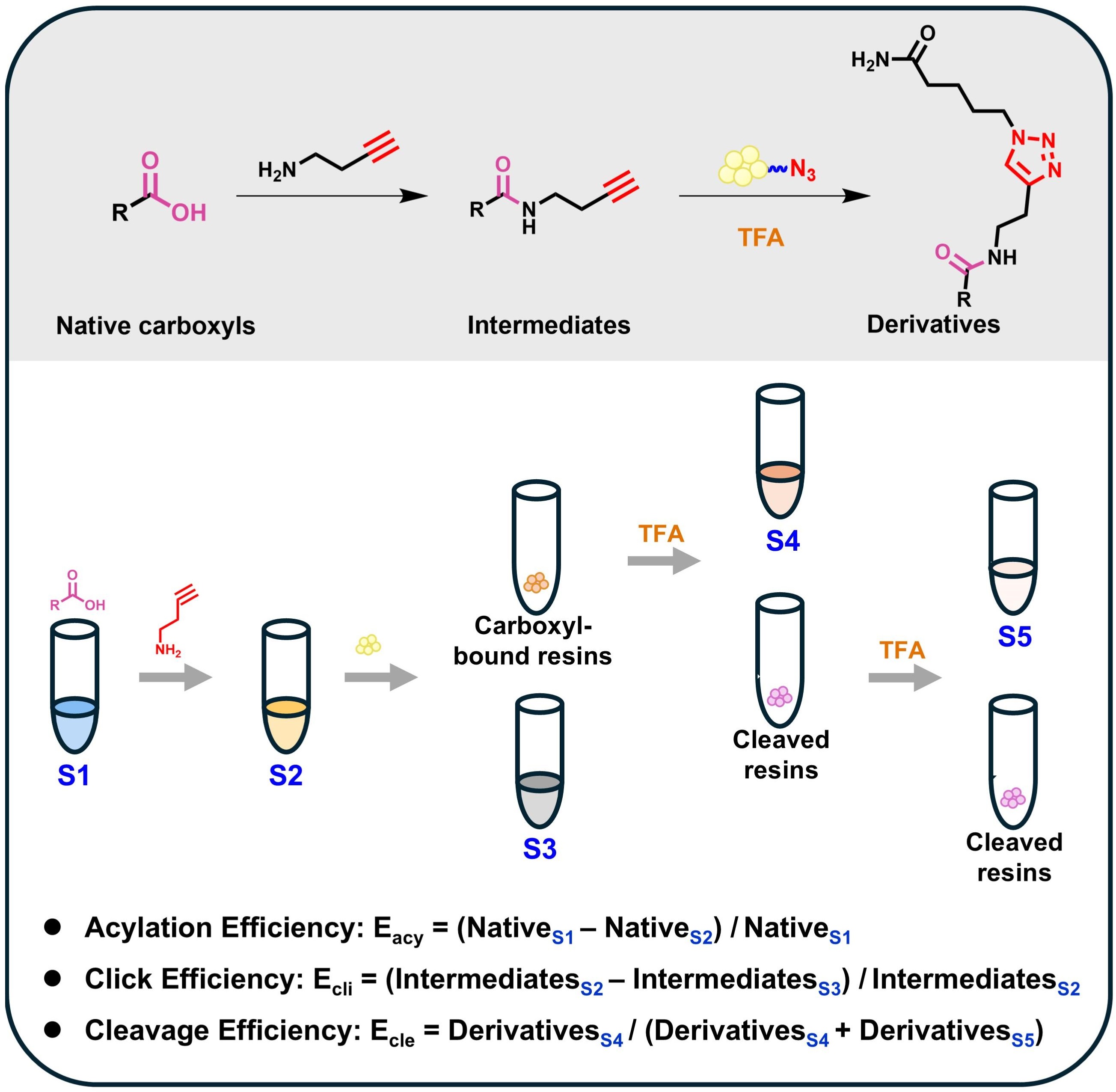


**Figure S7.** Workflow for calculating reaction efficiency. Taking carboxyl metabolites as an example, reaction efficiencies were determined based on peak intensities measured before and after each reaction step. Acylation efficiency (Step 1) was evaluated by quantifying the remaining native carboxyl standards in negative ion mode from the unreacted (S1) and reacted (S2) mixtures. Click chemistry efficiency (Step 2) was assessed by estimating the remaining intermediates in positive ion mode in the unreacted (S2) and reacted mixtures (S3). Cleavage efficiency was calculated as the ratio of derivatives in S4 to the total derivatives in S4 and S5.


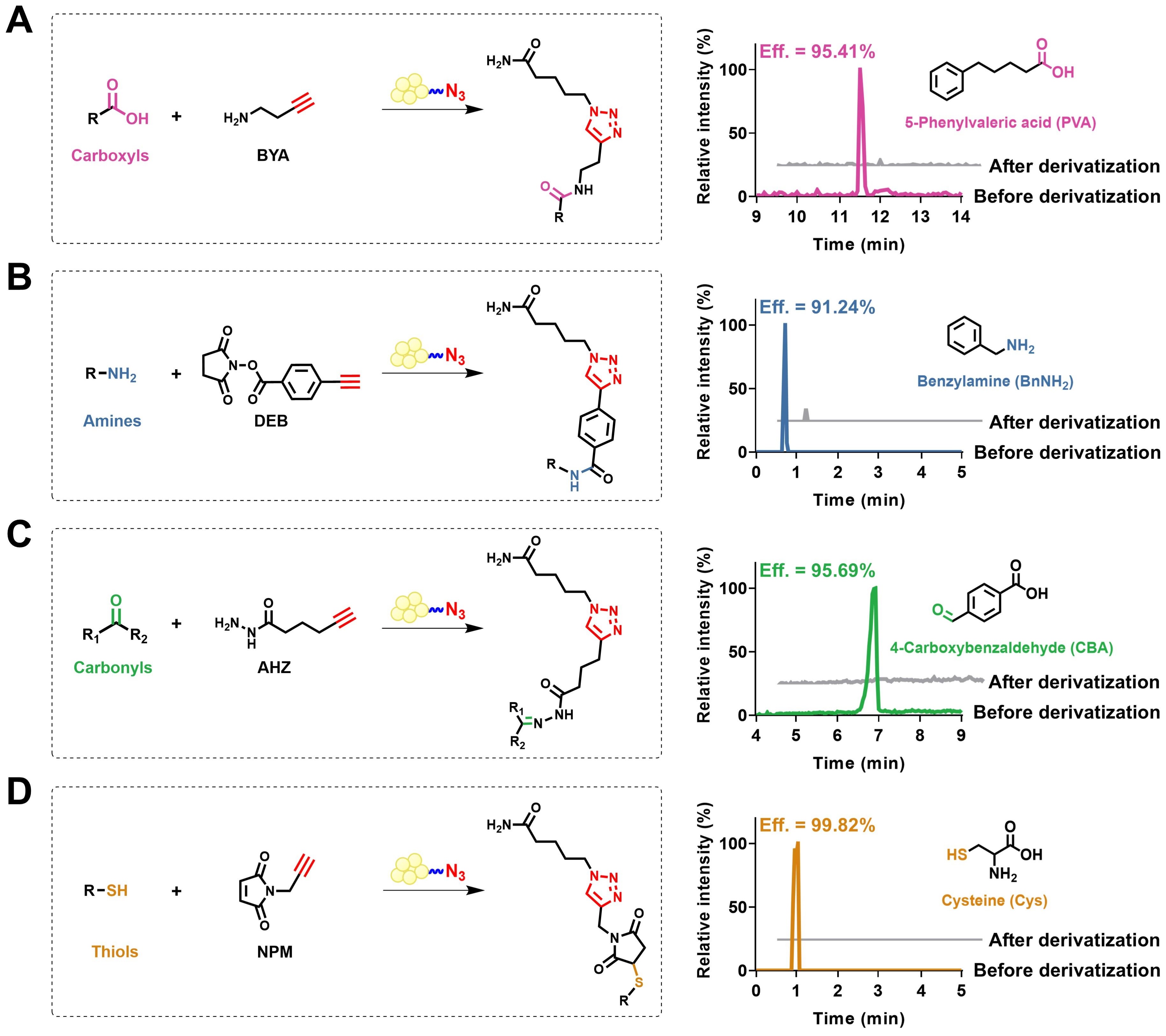


**Figure S8.** Extracted ion chromatograms (EICs) of representative (A) carboxyl, (B) amine, (C) carbonyl, and (D) thiol standards before and after derivatization, showing the residual native signals after derivatization. Eff., Reaction efficiency.


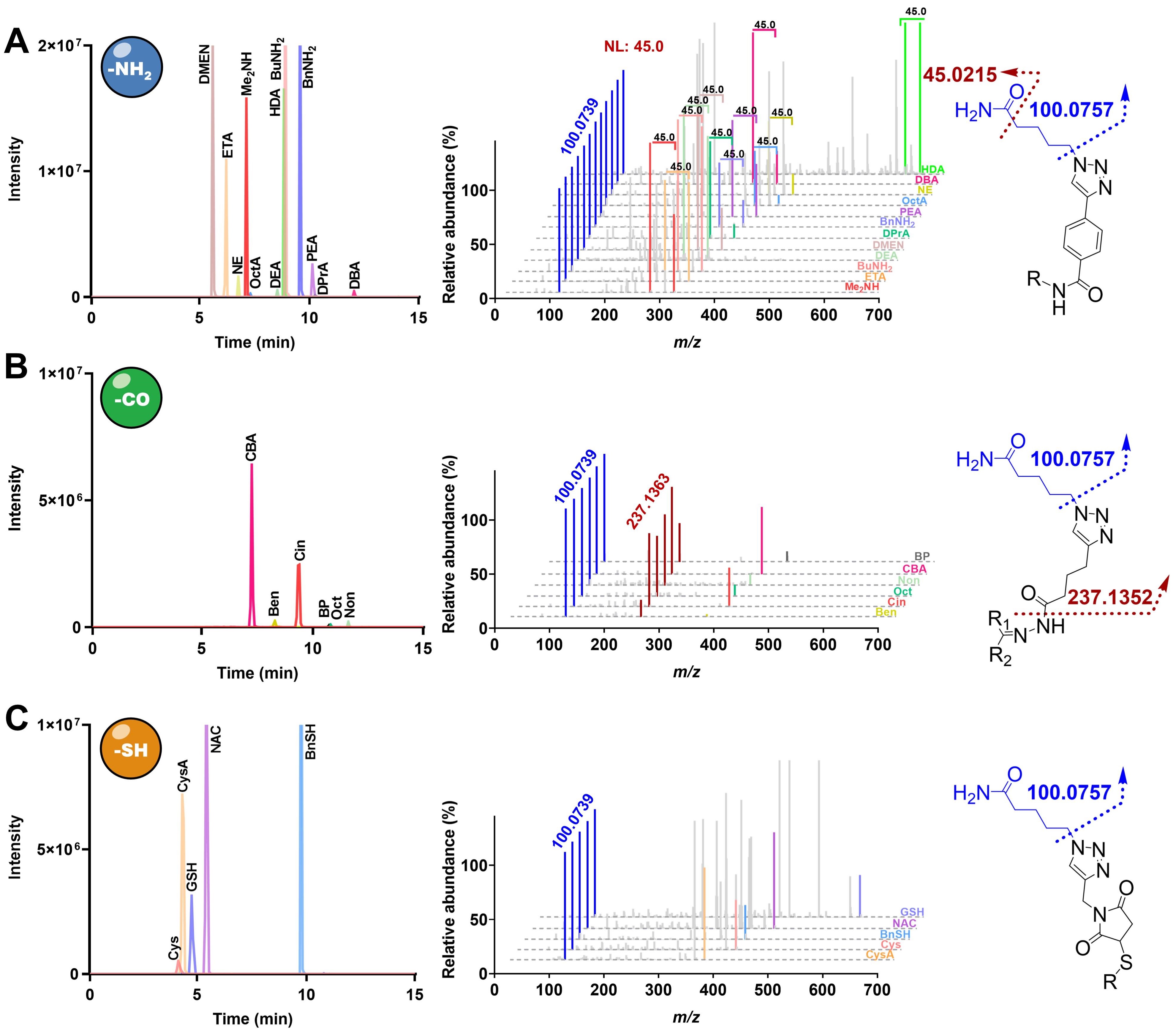


**Figure S9.** Extracted ion chromatograms (EICs, left), MS/MS spectra (middle), and proposed fragmentation pathways (right) of representative (A) amine, (B) carbonyl, and (C) thiol metabolite derivatives detected in the positive ion mode. The abbreviations of standards correspond to those shown in Figure S1.


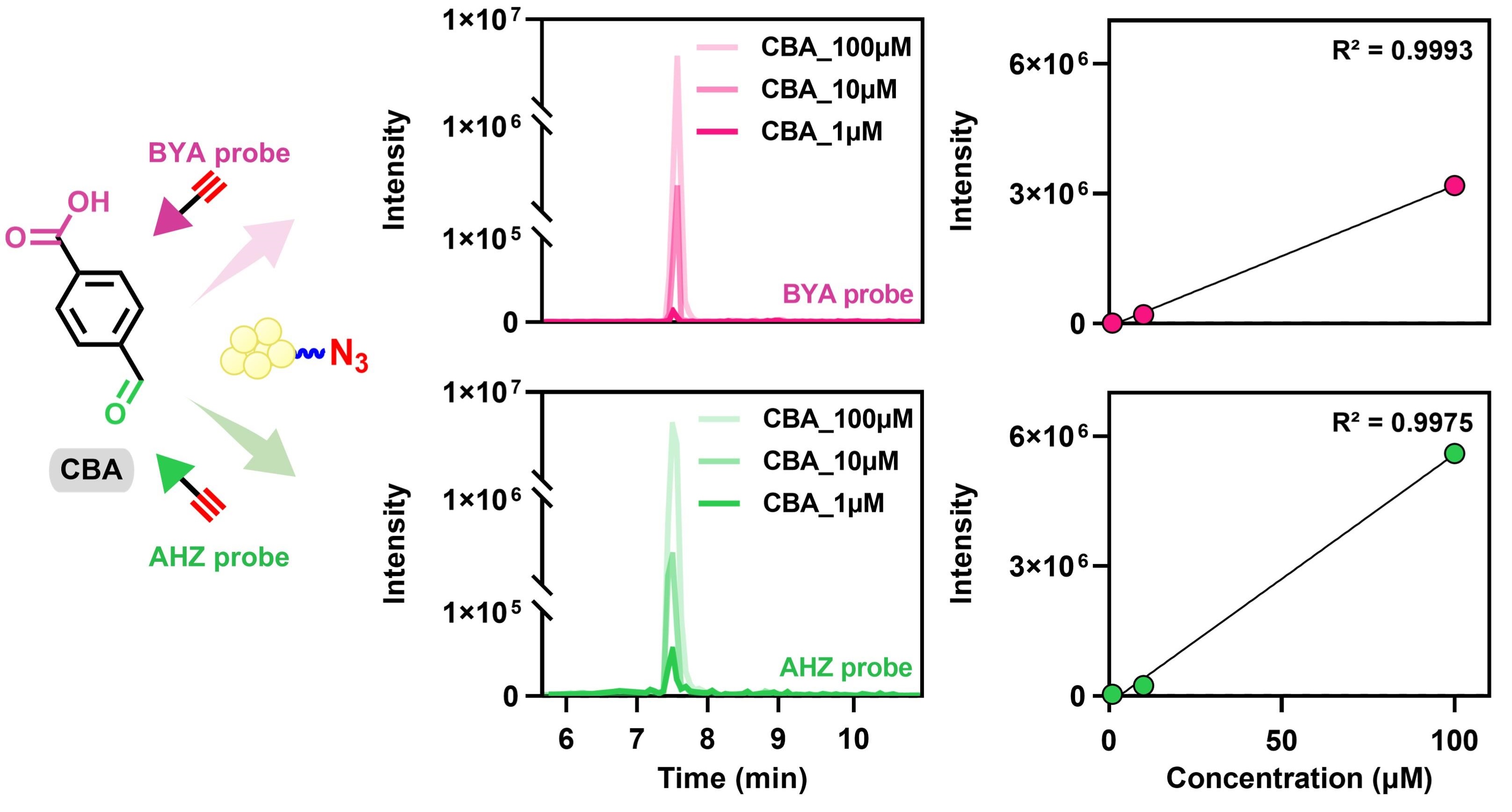


**Figure S10.** Extracted ion chromatograms (EICs) of 4-carboxybenzaldehyde (CBA) derivatives obtained at different initial concentrations (1, 10, and 100 μM), following derivatization with carboxyl‑reactive and carbonyl‑reactive probes.


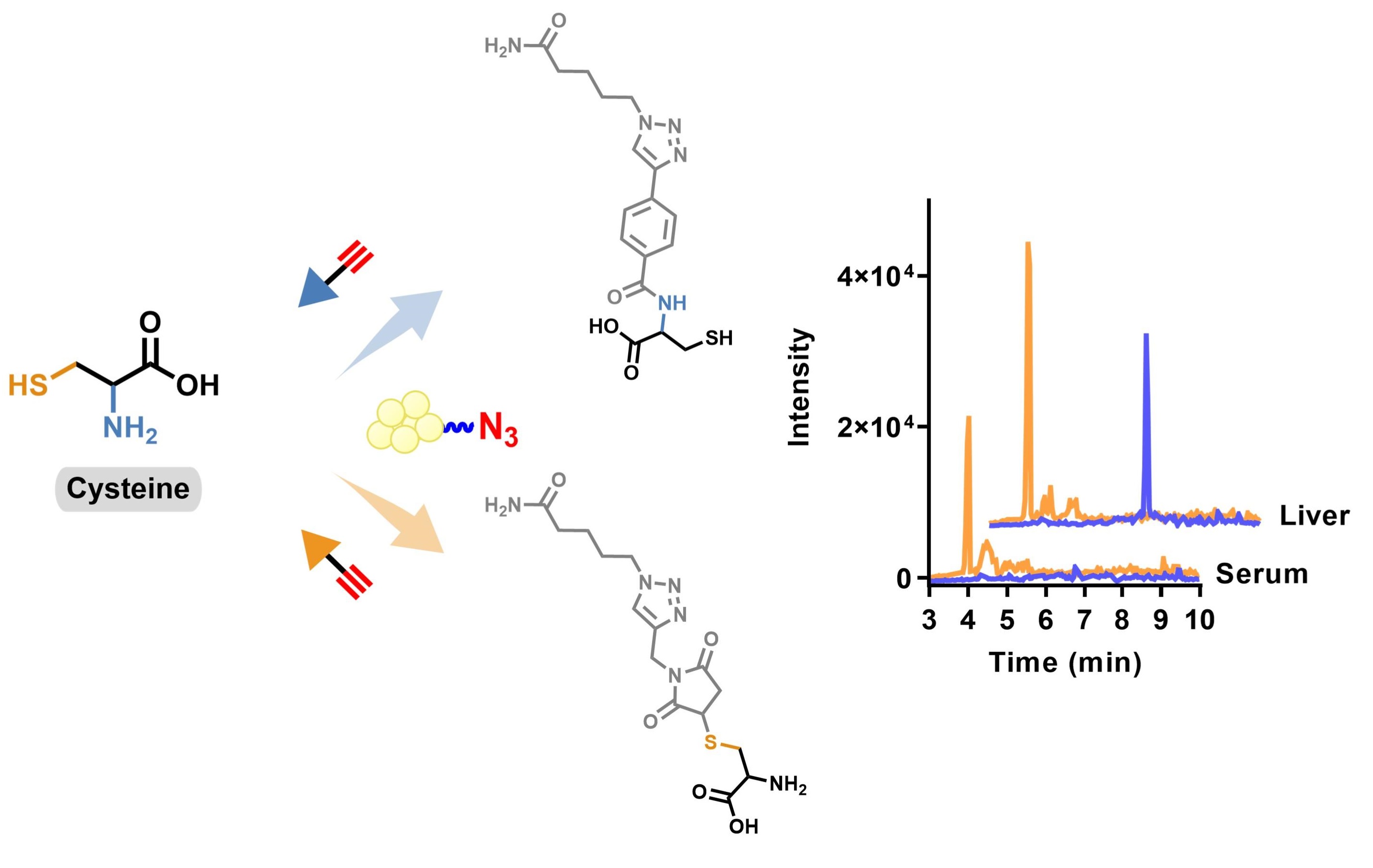


**Figure S11.** Extracted ion chromatograms (EICs) of cysteine derivatives obtained in different biological samples following derivatization with amine‑reactive and thiol‑reactive probes.


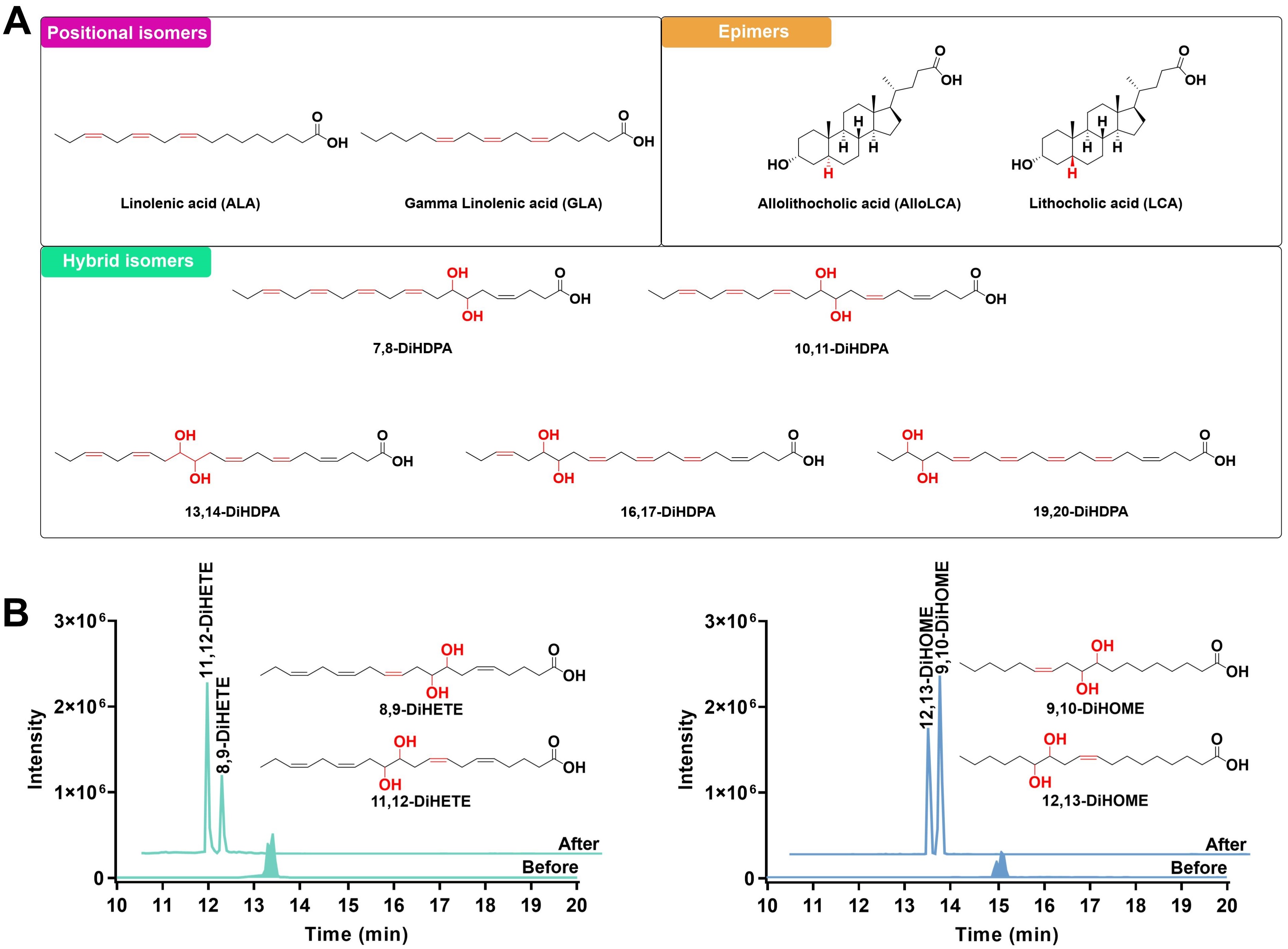


**Figure S12.** (A) Chemical structures of representative carboxyl isomers and **(**B) their chromatographic behavior before and after MREP.


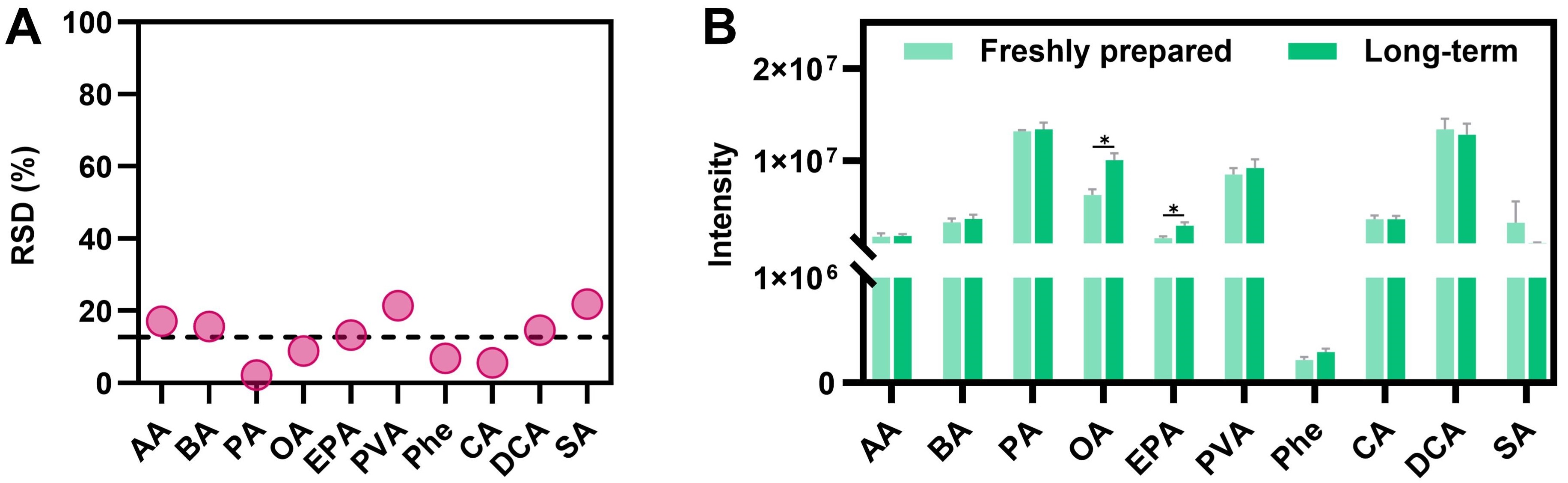


**Figure S13.** Evaluation of batch-to-batch variation and long‑term storage stability of the ACER resin using ten representative carboxyl metabolites. (A) Relative standard deviations (RSD) obtained from three independently prepared batches (n = 3). (B) Comparison of signal responses between freshly prepared resin and resin stored for approximately one year (n = 3). All data were expressed as the mean ± SEM, with **p* < 0.05, and p values were from Student’s *t*-test.


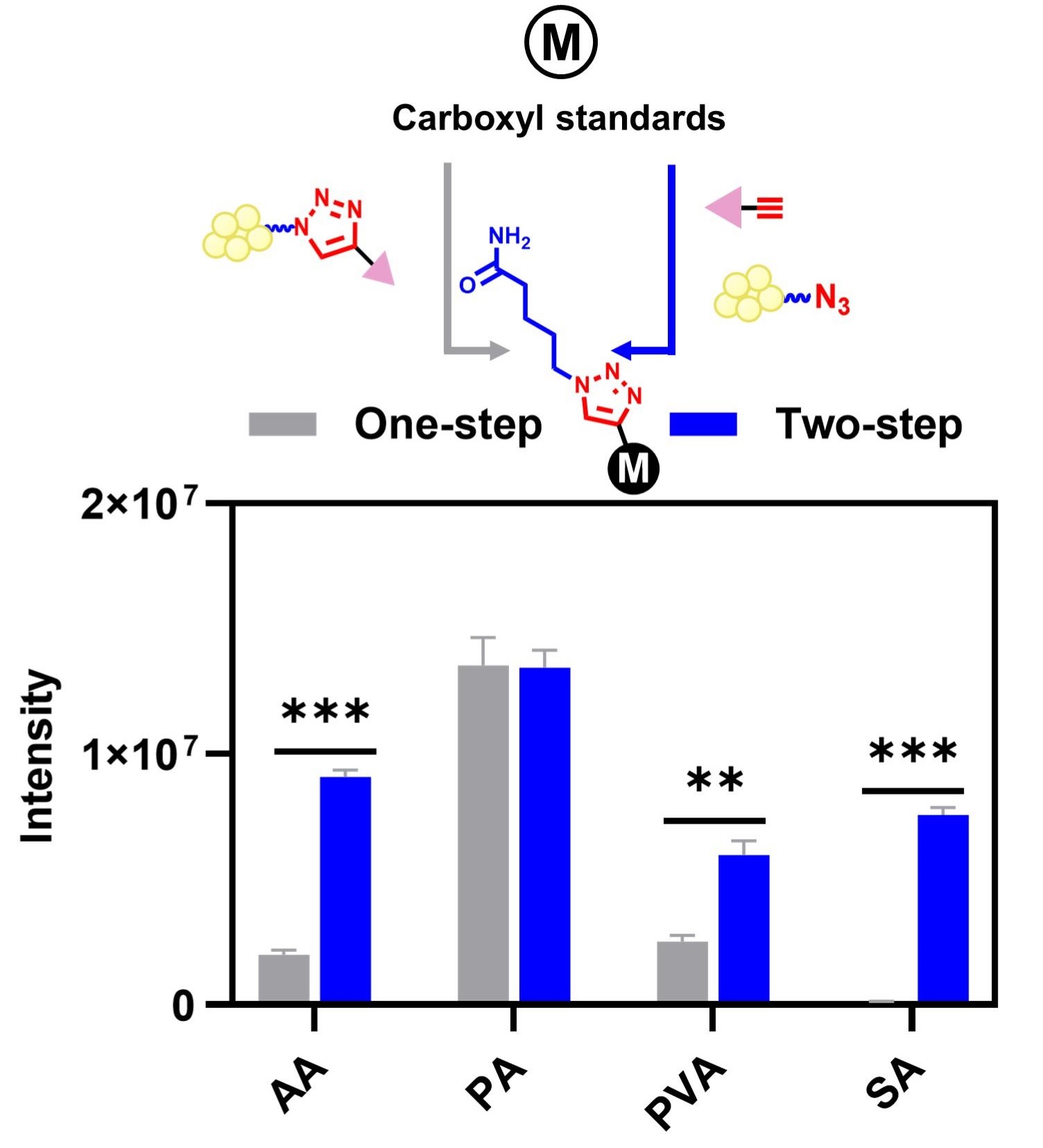


**Figure S14.** Comparison of one-step and two-step strategies using representative carboxyl standards (n = 3), all data were expressed as the mean ± SEM, with ***p* < 0.01, ****p* < 0.001, and p values were from Student’s *t*-test.


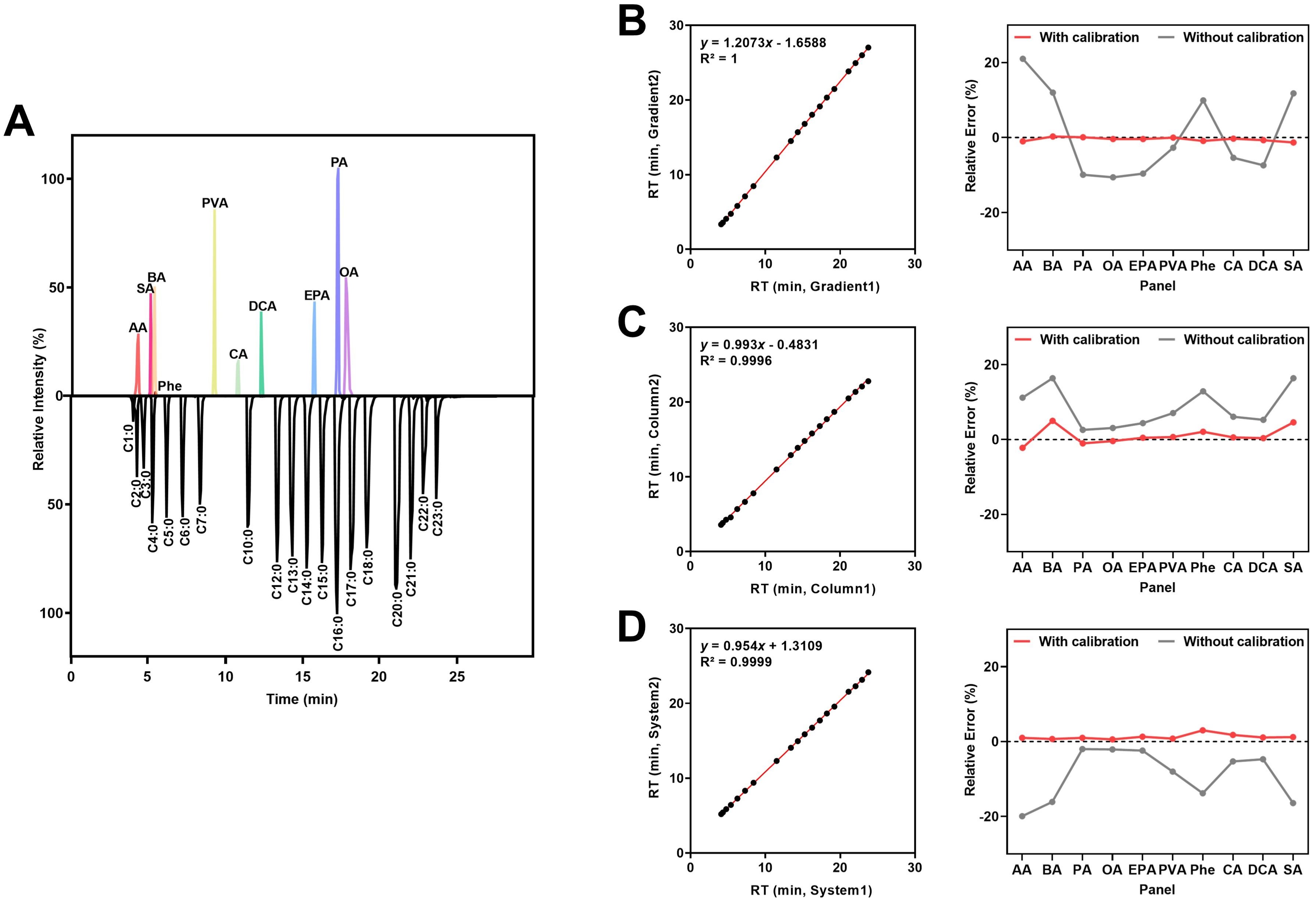


**Figure S15.** Retention time (RT) correction using derivatized saturated fatty acids (SFAs). (A) Extracted ion chromatograms (EICs) of derivatized carboxyl standards (top) and derivatized SFAs (bottom). (B-D) Regression analysis (left) of RT values from the derivatized standards under different **(**B) gradient conditions, (C) chromatographic columns, and (D) LC systems. The corresponding errors with (red) and without (grey) RT correction using derivatized SFAs are shown on the right. The chromatographic conditions are detailed in Table S4.
